# Catalytic Innovations in the Aza-Michael Reaction: An Experimental Benchmarking Focused on Sustainable Approaches

**DOI:** 10.3390/molecules30132674

**Published:** 2025-06-20

**Authors:** Silvia Izquierdo, Carlos J. Durán-Valle, Pedro Cintas, Ignacio M. López-Coca

**Affiliations:** 1 Department of Organic and Inorganic Chemistry, School of Technology, University Research Institute for Sustainable Territorial Development (INTERRA), Universidad de Extremadura, 10003 Cáceres, Spain; sizquierdo@unex.es; 2 Department of Organic and Inorganic Chemistry, Faculty of Sciences, University Institute for Water Research, Climate Change and Sustainability (IACYS), Universidad de Extremadura, 06006 Badajoz, Spain; carlosdv@unex.es

**Keywords:** aza-Michael reaction, catalysis, hydrothermal carbon, K10 montmorillonite, ionic liquids, green chemistry

## Abstract

This study explores a series of eco-compatible, safe, inexpensive, and recyclable catalysts for the aza-Michael reaction, an essential transformation for constructing C-N bonds. In particular, we focus on hydrothermal carbons (HCB and HCC) prepared from chestnut cupule waste under mild, aqueous conditions, offering a sustainable alternative to traditional pyrolytic methods. These carbonaceous solids, thoroughly characterized by physicochemical techniques, exhibit notable catalytic activity, completing aza-Michael reactions in as little as 5–30 min for various model substrates. Their performance was benchmarked against Montmorillonite K10, [Cho][Pro] ionic liquid, and K10+[Cho][Pro], with the latter combination and [Cho][Pro] alone giving the fastest conversions. For example, the reaction of benzylamine with acrylonitrile reached complete conversion (typically 95% yield) in five minutes using [Cho][Pro], K10+[Cho][Pro], or likewise with HCB and HCC. Although the reactions catalyzed by hydrothermal carbons did not outperform in general those using K10-[Cho][Pro] or [Cho][Pro], they proceeded rapidly and afforded good to excellent yields. Furthermore, the HCC catalyst demonstrated excellent recyclability, maintaining its activity and yield over at least five cycles. These findings underscore the potential of hydrothermal carbons as efficient green heterogeneous catalysts, combining high surface area, porosity, and reusability with strong catalytic performance and scalability, in alignment with the principles of the circular economy.

## 1. Introduction

For more than two decades, the practice of chemistry has been progressively integrated into a sustainable agenda, with current portfolios focusing on renewable feedstocks, catalytic approaches, and greener reaction media [1,2,3]. Merging biomass residues and catalysis for producing added-value compounds and commodity chemicals has gained significant attention in this context [4]. Among the plethora of methods for constructing C-N bonds, the aza-Michael reaction exhibits a competitive rivalry with other existing protocols due to its simplicity and versatility, allowing for the straightforward preparation of diverse nitrogen-containing compounds with applications ranging from agrochemicals to pharmaceuticals and polymers [5,6,7,8]. Both intramolecular and asymmetric variations have also been developed in recent years as well [9,10,11].

The aza-Michael reaction, which involves the conjugate addition of a nitrogen nucleophile to an α,β-unsaturated carbonyl compound, can be conducted in the absence of catalysts; however, it is often sluggish and can take a considerable amount of time to complete. Catalytic variations are clearly advantageous in the search for faster and more selective transformations, leaving aside that this transformation is highly dependent on steric and electronic effects. Matched combinations of nucleophilic amines and highly electrophilic alkenes, free from steric hindrance, typically yield optimal results. A broad range of catalysts has been employed with variable success, having both pros and cons, including Brönsted and Lewis acids, solid and supported reagents, and ionic liquids (ILs) [5,6,9,10,11,12,13,14,15,16,17,18,19,20,21,22,23]. The use of ionic liquids (ILs) and/or deep eutectic solvents is particularly attractive, as these substances often serve as both solvents and catalysts [24]. An interesting family of biocompatible ILs is provided by the interaction of choline and amino acids [25,26,27,28,29]. In a recent study, we demonstrated the efficiency of cholinium amino carboxylates in enhancing aza-Michael reactions through experimental and theoretical analyses [30,31]. Computation reveals that the catalyst lowers significantly the activation barrier leading to C-N bond formation through charge stabilization at the saddle point, which is in turn associated with a dual cooperativity, i.e., H-bonding and conformational restriction, especially with proline, [Cho][Pro]-catalyst, due to a less flexible side chain.

In light of our current motivation to develop greener synthetic routes, we report herein the alternative use of carbonaceous catalysts derived from biomass residues, which are renewable, metal-free, and abundant materials. This not only reduces the dependence on fossil resources but also mitigates the waste disposal issues associated with biomass remnants. In particular, the present study focuses on hydrothermal carbons (HCs), which are now positioned at the forefront of activated carbons and charcoals for numerous applications. Hydrothermal carbonization involves the conversion of biomass residues under high-pressure, high-temperature conditions, resulting in the formation of carbonaceous materials with unique properties [32,33]. Their porous structure and large surface area provide ample active sites for catalysis, facilitating efficient adsorption of reactants and subsequent product formation. Additionally, their tunable surface chemistry allows for modification and optimization of their catalytic performance. Accordingly, two inexpensive biomass-based HCs have been evaluated in a series of representative aza-Michael reactions, and their catalytic efficiency has been compared with that of other sustainable catalysts, namely montmorillonite K10 [34,35], a well-known naturally occurring clay mineral, and the aforementioned cholinium prolinate IL, as well as their combination. All the structural features of the solid catalysts have been thoroughly evaluated using physicochemical methods to unveil their morphology and ensure reproducibility.

## 2. Results and Discussion

**Figure 1:**
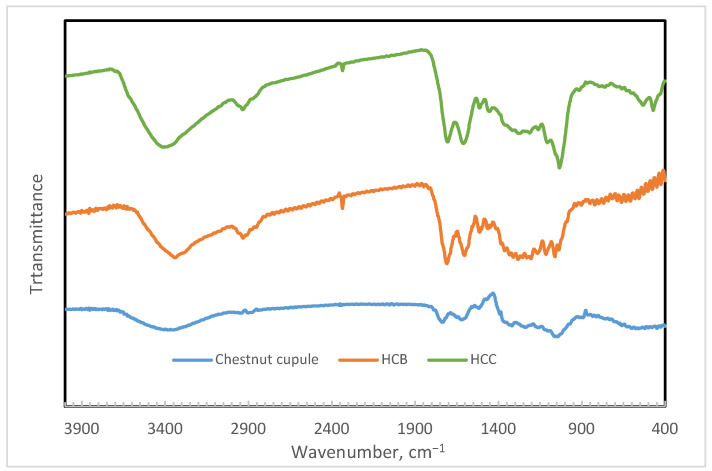
FTIR spectra of hydrothermal carbons HCB and HCC, and chestnut cupule. The plots for hydrothermal carbons are reproduced from Ref. [36], an open-access article, under the CC-BY license.

Hydrothermal carbonization has been applied to chestnut cupule residues, a typical waste product from sweet chestnuts, a seasonal autumn fruit abundant in the Northern Hemisphere and Mediterranean countries. In a recent study, we also reported that chestnut cupules can be converted to hydrothermal carbons and used as adsorbents for PFAS (polyfluorinated alkanes) in polluted water [36,37]. Following the same protocol, finely powdered chestnut cupules with average sizes of 0.5–1 mm and 1–2 mm, hereafter referred to as HCB and HCC, respectively, were hydrothermally carbonized (see Experimental). A preliminary analysis by Fourier transform infrared (FTIR) spectroscopy of hydrothermal carbons HCB and HCC and chestnut cupules (Figure 1) reveals a large number of functional groups, which include aliphatic O–H, C–H, C=O, and C–O bonds. The most intense broad absorption centered at 3400 cm^−1^ corresponds to the stretching band of the O–H group, present in either alcohol or carboxylic acid functionalities. The band below 3000 cm^−1^ is clearly indicative of aliphatic C–H bonds, as the corresponding stretching band for aromatic C–H bonds usually appear above that wavenumber. The band near 1700 cm^−1^ can be attributed to carbonyl (C=O) bonds, whereas the neighboring absorption at 1610 cm^−1^ is consistent with both C=C and C=N bonds in conjugated systems. Bands between 1500 and 1300 cm^−1^ should be attributed to the carbon backbone of these materials and most likely involve bending bands of O–H and C–O bonds. Likewise, absorptions between 1300 and 1000 cm^−1^ presumably include asymmetric and symmetric vibrations of C–O and C–N bonds. Overall, FTIR data point to low levels of graphitization, as witnessed by the high content of oxygen- and hydrogen-containing functional groups.

**Figure 2:**
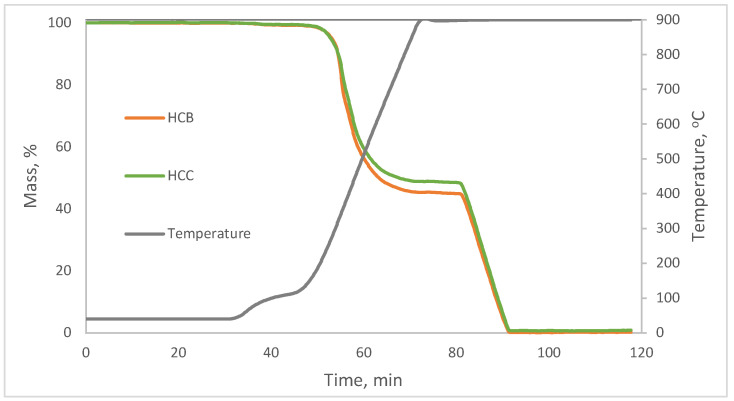
Thermal analysis of chestnut cupules.

Moreover, the thermogravimetric (TG) analysis of chestnut cupules (Figure 2) indicates that most cupules are made up of volatile matter, which usually contains a large number of heteroatoms. The fixed carbon content is low, as is usually the case of lignocellulosic biomass, and a very low ash content stands out too [38]. On the other hand, the TG profile shows that the degradation of volatile matter begins around 50 min, after reaching 200 °C. In order to maintain a high proportion of oxygenated functional groups, it is convenient not to exceed this temperature.

Since, for comparative purposes against hydrothermal carbons, the natural aluminosilicate montmorillonite K10 has been assessed in aza-Michael reactions, characterization of the latter, and specifically the commercial material employed, is compulsory. Owing to its intrinsic acidity, attributed to a high density of Brønsted and Lewis acid sites, K10 montmorillonite exhibits exceptional efficacy in catalytic applications [34,39]. Its distinctive layered arrangement, decorated by exchangeable cations and abundant hydroxyl groups, constitutes a suitable framework for multiple reactions [34,35]. The interlayer spaces accommodate numerous guest molecules, facilitating adsorption, diffusion, and subsequent catalytic conversion. The use of clays is invariably problematic unless the surface characteristics are specified. When using neat K10, variable results were obtained from batch to batch. As the supplier could not warrant a constant chemical composition for an otherwise natural mineral, we characterized, in detail, the material employed in our catalytic tests using various standard techniques such as nitrogen adsorption isotherm, point of zero charge (PZC), porosimetry, scanning electron microscopy (SEM), energy dispersive X-ray (EDX) analysis, powder X-ray (PXR) diffractometry, X-ray photoelectron spectroscopy (XPS), and wavelength-dispersive X-ray fluorescence (WDXRF).

**Figure 3:**
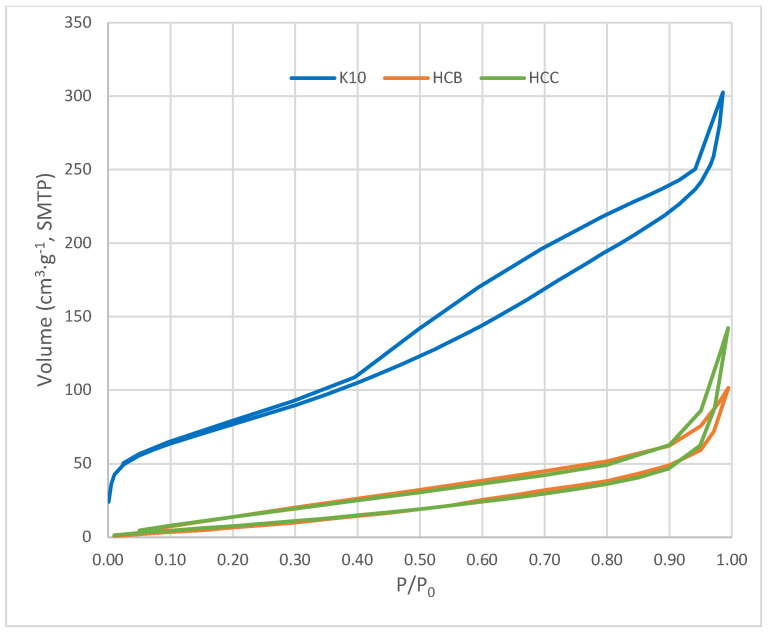
Isotherms of N_2_ adsorption obtained at 77 K.

The nitrogen adsorption isotherms (Figure 3) exhibit distinct characteristics for the two types of materials. The isotherms of HCB and HCC correspond to type III according to the IUPAC classification [36,37], indicating the absence of micropores. In contrast, the isotherm of K10 is type IV, with an H4-type hysteresis cycle and a small amount of micropores.

Textural characterization using the conventional BET (Brunauer–Emmett–Teller) method [40] revealed a specific surface area of 279.5 m^2^·g^−1^ for K10, whereas the S_BET_ values for hydrothermal carbons derived from chestnut cupules ranged between 42.3 and 53.2 m^2^·g^−1^ [36]. Micropore volume, determined using the Dubinin–Radushkevich method, was significantly higher in K10 (0.107 cm^3^·g^−1^) compared to the hydrothermal carbons (0.007 cm^3^·g^−1^). In contrast, mesopore volume, measured by mercury porosimetry, showed only minor differences, with values of 0.078 cm^3^·g^−1^ for K10 and 0.061 cm^3^·g^−1^ for both HCB and HCC. For clarity, tabulated data have been included in Table 1. Hydrothermal coals have not undergone an activation process that would allow porosity, especially sufficient microporosity to be developed. This type of porosity is mainly responsible for the specific surface of carbonaceous materials, which, in turns, accounts for the low surface specific area of such materials.

**Figure 4:**
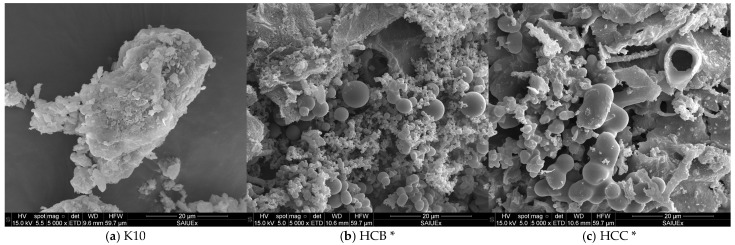
SEM images at 5000× of K10 (**a**), HCB (**b**), and HCC (**c**). * Reproduced from Ref. [36], open-access article under the CC-BY license.

Scanning electron microscopy (SEM) images of the catalysts were recorded, with three representative images presented in Figure 4. K10 exhibits irregular particles of varying sizes and aggregation states. In contrast, the hydrothermal carbons retain structural features of the original plant material, particularly large voids arising from the cellular substructure. Further details are included in the Supplementary Information.

The X-ray diffraction (XRD) pattern of K10 (see Supplementary Information) was analyzed to confirm its structure, yielding results consistent with previously reported data [41,42]. It is noteworthy that native K10 and K10/IL ionogels and composites show some reflections matching our data. Thus, peaks observed at 2θ values 8.89, 17.81, 19.85, and 35.04 could be ascribed to (001), (002), (110), and (101) lattice planes [43,44].

Wavelength dispersive X-ray fluorescence (WD-XRF) analysis of K10 revealed the predominant presence of Si > Al > Fe > K > Mg, alongside oxygen (Table 2).

The results obtained by EDX analysis are consistent with those from XRF, confirming that the most abundant metallic elements are Si > Al > Fe > K > Mg. Additionally, carbon was detected, an element not analyzable by WDXRF, likely due to sample contamination from adventitious carbon. Elemental mapping reveals a broad distribution of all detected elements, though certain regions appear enriched in specific element pairs, such as Al and K or Ca and C. In contrast, elements like Ti and Na are localized, while Fe, O, and Mg exhibit a more uniform distribution (See Supplementary Information). The elemental composition of HCB and HCC, determined by elemental analysis, is presented in Table 3. As expected, these materials are rich in carbon, though their carbon content is lower than that of other carbonaceous materials. Notably, their high oxygen content suggests the presence of a substantial number of oxygenated functional groups. Additionally, the ash content is relatively low.

Biomass typically has 40–50% oxygen by mass. If a pyrolysis treatment is carried out exceeding 300–350 °C, the char obtained contains only 5% oxygen. For this reason, when a high content of functional groups is desirable, hydrothermal carbonization at moderate temperatures constitutes a good choice [45]. In general, hydrothermal coals obtained from simple carbohydrates under such conditions usually have a carbon content between 65% (hexoses) and 69% (pentoses) [46]. Since a large part of the biomass is made up of two polysaccharides (cellulose and hemicellulose), it is to be expected that this behavior will be maintained. On the other hand, materials obtained by pyrolysis and activation processes at high temperatures have a lower proportion of heteroatoms and therefore a greater amount of carbon. For comparative analysis, Table 4 collects previous data from the literature showing the measured carbon contents of different carbonaceous materials.

The abundance of oxygenated functional groups should be imparting strong acidity to these solids. Table 5 presents the corresponding point of zero charge (PZC) values. Moreover, and, once again, for comparative assessment, Table 6 summarizes the values obtained for other acidic carbons, including hydrothermal ones.

The PZC value depends on the starting sample, although, in general, it can be indicated that commercial, unmodified activated carbons have alkaline PZCs. The adsorption of a metal oxide (N-Zr) appears to have some influence, but the use of strong acids does it to a greater extent. Thus, it can be seen in Table 6 that the use of nitric acid decreases the PZC between 2.6 (M-N) and 3.8 (C-X-N) units. This is mainly due to the formation of carboxyl groups. The effect is greater when sulfuric acid is used, although in some cases the number of acid groups is lower than in the presence of nitric acid. This can be attributed to a greater strength of the acid functional groups, as sulfonic groups are formed. Regarding hydrothermal coals (HT), these materials are acidic because they retain a large amount of functional groups from the biomass. And this acidity increases (HT-S) when sulfuric acid is employed.

As a sensitive and non-destructive surface-monitoring technique, X-ray photoelectron spectroscopy (XPS) provides further insight into the surface composition and chemical functionality of the catalytic systems. Thus, XPS data show a higher content in carbon and, conversely, a lower estimation of oxygen relative to those obtained by elemental microanalysis (Table 7). This result suggests that hydrothermal carbonization affects the outer zone of chestnut cupules to a greater extent than it does on the inner core, which still preserves a significant fraction of oxygenated functional groups.

In general, peak energies support the chemical nature of functionalities assessed by FTIR spectra. For both HCs only one broad peak could be observed for C 1s at ~285.9 eV plus a dominant O 1s peak at 533.5 eV. Unfortunately, the N 1s spectrum exhibited a low signal-to-noise relationship to allow us a clear-cut distinction of assignable peaks. The energy measured for both C 1s and O 1s peaks are consistent with the prevalence of both C−O bonds, which can be ascribed to ether or alcohol linkages, and carbonyl (C=O) groups on the carbonaceous surface. The fact that no C 1s signals near 299 eV could be detected rules out the presence of more oxidized carbons atoms, such as ester or carboxylic acid bonds.

As already indicated and in line with our current research pursuits, we have screened the potentiality of the above-mentioned catalysts and other variations to archetypal aza-Michael reactions. This textbook-knowledge and atom-economical transformation has gained considerable interest in green chemistry agendas [53]. As noted in the introductory observations as well, the reaction usually works well with unhindered substrates, even in an uncatalyzed fashion. It can be run in different solvents or solventless conditions, and often, thermal activation affords high conversion of the corresponding Michael adducts in less than 1 h.

**Scheme 1:**
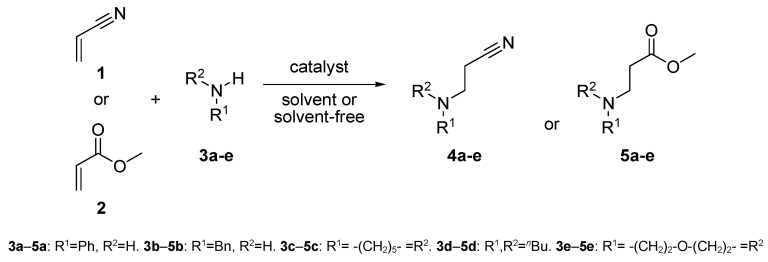
Representative aza-Michael reactions with acrylates and amines.

Scheme 1 shows the general strategy used herein, where either methyl acrylate or acrylonitrile were chosen as representative Michael acceptors, while a series of monosubstituted or disubstituted aliphatic amines plus aniline were employed as Michael donors. The resulting adducts are obviously the products of 1,4-conjugate additions, even if other byproducts can eventually be detected or isolated, such as amides arising from the competitive nucleophilic acyl substitution.

Results are shown in Table 8. Reactions were forced to completion, as inferred from TLC analysis (i.e., disappearance of the olefinic partner, limiting reagent), from which essentially quantitative yields of adducts were obtained. In general, all reactions were fast at room temperature (5–30 min), although in some cases, the aza-Michael reaction was incomplete after 3 h, yet the corresponding product could be isolated in good yield. The TLC protocol is sensitive enough as a few μg/spot represents a valuable threshold. Although reactions could even be faster, we were not interested in precise kinetic analysis to determine reaction rates but rather to detect complete transformations under catalytic conditions as mild as possible without further activation.

Data gathered in Table 8 evidence that cholinium prolinate was the most efficient catalyst in terms of reaction times, as unveiled in our previous study [30], although similar results could be achieved with hydrothermal carbons. Slower transformations were obtained when using the K10 aluminosilicate alone, which improved by impregnation with the ionic liquid. Given the viscosity of the latter, and the heterogeneous character of the other catalytic systems, mass transfer clearly represents a bottleneck. This could explain why some reactions did not go to completion, a fact also ascribed to steric factors in the case of dibutylamine.

To assess the catalytic efficiency in the studied reactions, experiments were conducted using methyl acrylate under catalyst-free conditions. The results indicate that, overall, the presence of the catalyst enhances the reaction rate to varying extents, ranging from moderate to substantial acceleration. However, in certain cases, no significant improvement was observed compared to the uncatalyzed reaction (entries 7, K10, and 8, K10+[Cho][Pro]), likely due to dilution effects or catalyst-induced scavenging in reactions that proceed rapidly in the absence of a catalyst.

Catalyst recyclability is a key parameter in evaluating the efficiency and sustainability of a catalytic system. In our previous work, we demonstrated that the ionic liquid [Cho][Pro] could be reused for at least five consecutive cycles without any observable loss of activity. Building on these findings and the promising performance of the hydrothermal carbon catalyst (HCC), we investigated the reusability of HCC in the model aza-Michael reaction between piperidine and methyl acrylate at room temperature. Remarkably, HCC retained its catalytic activity over five successive cycles, consistently achieving complete conversion within five minutes. In all cases, the desired product was obtained in good, isolated yields ranging from 78% to 90%, confirming both the robustness and practical utility of this sustainable catalyst.

To further assess the practical applicability of HCC, a scale-up experiment was performed under the same model reaction conditions. In the presence of 150 mg of HCC, 12 mmol of the amine were reacted with 10 mmol of the alkene. Monitoring by TLC confirmed that the reaction reached completion within five minutes, and the desired adduct was obtained in quantitative yield.

A systematic study of catalyst loading was not performed; however, preliminary tests using double the standard amount of HCC (30 mg) did not lead to significant improvements in reaction time or yield. Using less than 15 mg was considered impractical due to limitations in accurately weighing such small quantities.

To contextualize these results, multiple factors must be considered beyond catalytic activity alone, including catalyst accessibility, cost-effectiveness, and practical application. Biomass waste-derived catalysts, such as HCB and HCC, offer significant advantages due to their local availability, low cost, and technical feasibility. These attributes make them highly promising as cost-effective, high-performance catalytic materials, providing a sustainable alternative to conventional options.

## 3. Experimental Section

### 3.1. Materials and Methods

Solvents of either HPLC or ACS grade were purchased from Scharlab (Barcelona, Spain) and Labbox (Barcelona, Spain). Reactants were purchased from Acros Organics (Geel, Belgium) and Montmorillonite K10 (with reported surface area 220–270 m^2^·g^−1^) from Sigma-Aldrich (Saint Louis, MO, USA). Sweet chestnut (*Castanea sativa*) cupules were collected from chestnut groves in Valle del Jerte, in Cáceres province, Spain.

NMR spectra were recorded on a Bruker (Billerica, MA, USA) Avance 500 MHz spectrometer using CDCl_3_ as a solvent, and tetramethylsilane as an internal standard. TLC was performed on silica gel plates coated with fluorescent indicator F254 from Merck KGaA (Darmstadt, Germany). Flash chromatography was performed on Merck 60 silica gel (230–400 mesh) [54]. Amino acid-derived IL [Cho][Pro] was obtained following the protocol described previously [55].

### 3.2. Synthesis of Hydrothermal Carbons

Chestnut cupules were allowed to dry naturally. They were subsequently ground and sieved, collecting two fractions: 0.5–1 mm (HCB) and 1–2 mm (HCC). Then, they were subjected to hydrothermal treatment in a duralumin-coated Teflon™ reactor. Distilled water (100 mL) was added to sieved cupule of the appropriate particle size (15 g) in a hydrothermal reactor. The reactor was closed, placed in an oven at 200 °C, and kept for 24 h. It was then removed from the oven and allowed to cool down. The hydrothermal carbon was filtered and washed with distilled water (ca. 200 mL) on a filter. Subsequently, it was dried in an oven at 110 °C for eight hours.

### 3.3. Characterization of Solid Catalysts

Textural characterization was performed by N_2_ adsorption isotherms at 77 K in a Quantachrome (Hook, UK) Autosorb iQ2-C Series equipment with prior degassing at 110 °C for 12 h, and the specific surface area was calculated by applying the BET method [40]. Porosimetry was performed in a Quantachrome Poremaster 60. Elemental analysis (C, H, N, S, O) was performed using a Leco (St. Joseph, MI, USA) CHMS-932 elemental analyzer. C, H, N, and S were analyzed, and the difference was assigned to ash and oxygen content. Ash quantification was carried out in a Netzsch (Selb, Germany) STA 449 F3 Jupiter thermobalance. The point of zero charge (PZC) values were determined using the method proposed by Valente Nabais and Carrott [56]. X-Ray diffractograms (XRD) were obtained using a Bruker D8 Advance equipment. A FEI (Hillsboro, OR, USA) field emission scanning electron microscope (SEM) Quanta 3D FEG, was used to explore the surface morphological characteristics of all samples. The sample analysis was performed under high-vacuum conditions with a secondary electron detector. The surface of all samples was coated with gold due to their low conductivity. Energy dispersive X-ray analyses (EDX) were recorded with the same equipment. Wavelength dispersive X-ray fluorescence (WD-XRF) analysis was conducted using an S8 Tiger equipment from Bruker. X-Ray photoelectron spectra (XPS) were recorded on a SPECS (Zürich, Switzerland) FlexPS-ARPES-E SPECS instrument using monochromatic Al Kα radiation at 1486.68 eV. The geological oxide bead preparation method was used. The K10 montmorillonite sample was calcined at 1000 °C for one hour, resulting in a mass loss of 13.3%, partly explained by the presence of adventitious carbon.

### 3.4. Typical Protocols for Catalyzed Aza-Michael Reactions

(a) Reactions with K10 and ionic liquid [Cho][Pro]

A solution containing the amine (1.2 mmol) and the alkene (1.0 mmol) was stirred at room temperature in the presence of K10 (15 mg) and the ionic liquid [Cho][Pro] (0.25 mmol) until the reaction reached completion, as determined by TLC. After completion, to remove the catalyst and excess amine, the mixture was dissolved in either DCM or Et_2_O and filtered through an Allihn-type filter tube (10 cm height × 2 cm diameter) with a silica gel bed. The filtrate was then evaporated under reduced pressure to obtain the pure product. Alternatively, for water-soluble amines, the reaction mixture could be diluted with water and extracted with either DCM or Et_2_O, followed by drying the organic phase over anhydrous MgSO_4_ and evaporating under reduced pressure to yield the pure product. In some cases, further purification was performed using flash chromatography. All compounds were identified by their NMR spectra, which matched those of known samples.

(b) Reactions with solid catalysts

A mixture of the amine (1.2 mmol) and the alkene (1.0 mmol) was stirred in the presence of the solid catalyst (15 mg), i.e., HCB, HCC, or K10, at room temperature until the reaction was complete, as confirmed by TLC. Upon completion, the product was isolated, purified, and characterized following the same procedures as stated before.

(c) Hydrothermal carbon-catalyzed reactions. Large-scale procedure

Scale-up test was performed by reacting piperidine (12 mmol, 1.022 g) with methyl acrylate (10 mmol, 0.861 g) at room temperature in the presence of HCC (150 mg), as a model reaction. The process, monitored by TLC, was completed within five minutes. The reaction mixture was diluted with DCM (50 mL) and washed with DI water (2 × 30 mL); the organic phase was recovered and filtered through an Allihn-type filter tube (10 × 2 cm) with a silica gel bed. DCM was then removed by evaporation under low pressure to give the pure product in quantitative yield.

(d) Hydrothermal carbon-catalyzed reactions. Recycling procedure

To assess the recyclability of HCC a mixture of the piperidine (12 mmol) and the methyl acrylate (10 mmol) was stirred in the presence of HCC (150 mg) at room temperature until the reaction was complete, as confirmed by TLC. Upon completion, the hydrothermal carbon was filtered off and washed with DCM (2 × 20 mL) and distilled water (2 × 20 mL); the organic layer was separated, dried over magnesium sulfate, filtered, and evaporated to afford the desired product. The catalyst was dried overnight in an oven at 80 °C and used again in subsequent cycles. This process was tested up to five times with no loss of catalytic activity, i.e., all reactions were completed in five minutes, and the product could always be isolated quantitively.

## 4. Conclusions

In summary, hydrothermal carbon catalysts (HCB and HCC) prepared from biomass waste (chestnut cupules) demonstrated strong catalytic performance in the aza-Michael reaction. Their efficiency was comparable to that of [Cho][Pro] and K10+[Cho][Pro], promoting full conversions typically in 5–30 min for a range of amines and Michael acceptors under mild, solvent-free conditions. These materials facilitated the reaction through adsorption and surface activation, exploiting their high surface area, tunable porosity, and chemical stability. Beyond catalytic performance, their reusability, ease of separation, and waste-derived origin make them highly attractive for green and sustainable chemistry.

K10 alone, as expected, functioned as a mild Lewis acid, typically requiring longer reaction times (mostly within 30–40 min). In contrast, the [Cho][Pro] ionic liquid, as demonstrated in our previous work [30], accelerates the reaction through hydrogen bonding and electrostatic interactions, thereby stabilizing the transition state and promoting an efficient C-N bond formation pathway. The enhanced activity of the K10+[Cho][Pro] system highlights a bifunctional synergistic effect, where the ionic liquid activates the nucleophile and electrophile while K10 contributes surface acidity.

Altogether, this work demonstrates that biomass-derived hydrothermal carbons are viable, green catalysts for aza-Michael chemistry, representing a valuable step toward more sustainable catalytic methodologies.

## Figures and Tables

**Table 1:** Porosity and specific surface of catalysts.

Catalyst	Specific Surface ^a^ m^2^ g^−1^	V_micro_ ^b^ cm^3^ g^−1^	V_meso_ ^c^ cm^3^ g^−1^
K10	279.5	0.107	0.078
HCB	42.3	0.007	0.061
HCC	53.2	0.007	0.061

^a^ Brunauer–Emmet–Teller (BET) model. ^b^ Dubinin–Radushkevich model. ^c^ Mercury porosimetry.

**Table 2:** Composition of K10 by WD-XRF expressed as oxides.

Oxide	Concentration, %
SiO_2_	67.36
Al_2_O_3_	12.10
Fe_2_O_3_	3.05
K_2_O	1.70
MgO	1.37
TiO_2_	0.537
Na_2_O	0.29
CaO	0.221
ZrO_2_	0.0338
CuO	0.0131
ZnO	0.0106

**Table 3:** Elemental analysis of the hydrothermal carbons.

Sample	C, %	H, %	N, %	S, %	O, %	Ash, %
HCB	61.70	5.93	0.58	0.05	31.55	0.19
HCC	63.70	5.89	1.06	0.05	28.46	0.84

**Table 4:** Comparative analyses of carbon contents for different catalytic carbonaceous materials.

Material	C, %	Reference
Activated carbons prepared from mineral coal	>88	[47]
Activated carbon obtained from wood treated with nitric acid and activated with KOH	79	[48]
Activated charcoal obtained from coconut shells treated with nitric acid and activated with KOH	85–89	[48]
Commercial activated carbon	89–92	[49]
Acid modified activated carbon	83–87	[49]
Hydrothermal (from sucrose) carbon	64	[49]
Acid modified hydrothermal (from sucrose) carbon	60	[49]
Commercial activated fiber carbon	73	[50]
Modified fiber carbon	68–80	[50]

**Table 5:** Point of zero charge (PZC) measured for K10 and hydrothermal carbons.

Material	PZC
K10	3.5
HCB	3.8
HCC	4.8

**Table 6:** Point of zero charge of representative carbon catalysts, mostly with acidic surface properties.

Sample	Material Description	PZC	Reference
N	Commercial activated carbon	9.1	[49]
N-Zr	“N” with ZrO_2_	7.2	[49]
N-Zr-S1	“N-Zr” treated with H_2_SO_4_	4.9	[49]
N-HT	“N” + sucrose, hydrothermal treatment	9.2	[49]
N-HT-S	“N-HT” treated with H_2_SO_4_	4.4	[49]
M	Commercial activated carbon	10.0	[49]
M-N	“M” treated with HNO_3_	7.4	[49]
M-S	“M” treated with H_2_SO_4_	3.8	[49]
HT	Hydrothermal (from sucrose) carbon	2.8	[49]
HT-S	“HT” treated with H_2_SO_4_	1.9	[49]
C-X	Carbon xerogel	7.2	[51]
C-X-S	“C-X” treated with H_2_SO_4_	2.5	[51]
C-X-N	“C-X” treated with HNO_3_	3.4	[51]
N	Commercial activated carbon *	7.1	[52]
NS	“N” treated with H_2_SO_4_	2.8	[52]
NN	“N” treated with HNO_3_	3.8	[52]

* Sample different from the one indicated in the first line.

**Table 7:** Surface composition (% mass) of hydrothermal catalysts estimated from XPS data.

Catalyst	C 1s	O 1s	N 1s
HCB	71.83	27.39	0.79
HCC	71.30	27.45	1.25

**Table 8:** Aza-Michael reactions activated by green heterogeneous catalysts ^a,b^.

Entry	Alkene	Amine	Product	Catalyst						Yield ^c^
				HCB	HCC	K10	K10+[Cho][Pro]	[Cho][Pro] ^d^	--- ^e^	
				Reaction Time (min) ^f,g^						
1	**1**	**3a**	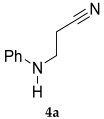	5	5	40	20	5		75
2	**1**	**3b**	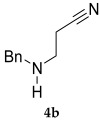	5	5	30	5	5		95
3	**1**	**3c**	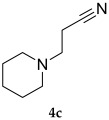	15	15	30	5	5		92
4	**1**	**3d**	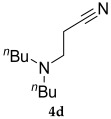	--- ^h^	--- ^h^	150	10	10		85
5	**1**	**3e**	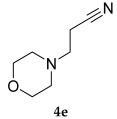	30	30	40	10	10		85
6	**2**	**3a**	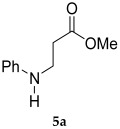	180	180	90	50	10	--- ^h^	70
7	**2**	**3b**	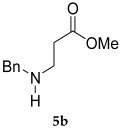	10	15	30	10	10	20	99
8	**2**	**3c**	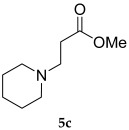	10	5	10	15	10	15	90
9	**2**	**3d**	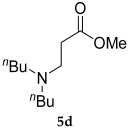	25	30	30	30	10	--- ^h^	78
10	**2**	**3e**	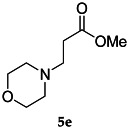	15	10	30	30	5	90	99

^a^ Reaction conditions: amine (1.2 mmol), alkene (1.0 mmol), catalyst (15 mg). ^b^ All reactions were conducted at room temperature (ca. 25 °C). ^c^ Representative yield (%), not optimized. ^d^ Results from our previous study [30]. ^e^ Neat reactions. ^f^ Time to completion, determined by TLC. ^g^ Products isolated in quantitative yield. ^h^ Incomplete reactions after 2 days.

## Data Availability

Supplementary Information is available in a separate file. Further data supporting this study are available from the corresponding authors upon request.

## Supplementary Materials

The following supporting information can be downloaded at https://www.mdpi.com/article/10.3390/molecules30132674/s1: Table S1: K10 porosimetry. Table S2: K10 Energy dispersive X-ray EDX. Table S3: K10 Wavelength-dispersive X-ray fluorescence WDXRF. Table S4: K10 X-ray photoelectron spectroscopy XPS. Table S5: K10 powder X-ray diffractometry PXR. Table S6. Thermogravimetric analysis (TGA) temperature program. Figure S1: K10 SEM images. Figure S2: K10 EDX elemental mapping. Figure S3: XPS Peak deconvolution for HCB and HCC. Figure S4. FTIR spectra for chestnut cupule, HCB, and HCC. Figure S5: ^1^H NMR [Choline][Proline]. Figure S6: ^1^H NMR 3-(phenylamino)propanenitrile (**4a**). Figure S7: ^1^H NMR 3-(benzylamino)propanenitrile (**4b**). Figure S8: ^1^H NMR 3-(piperidin-1-yl)propanenitrile (**4c**). Figure S9: ^1^H NMR 3-(dibutylamino)propanenitrile (**4d**). Figure S10: ^1^H NMR 3-morpholinopropanenitrile (**4e**). Figure S11: ^1^H NMR methyl 3-(phenylamino)propanoate (**5a**). Figure S12: ^1^H NMR methyl 3-(benzylamino)propanoate (**5b**). Figure S13: ^1^H NMR methyl 3-(piperidin-1-yl)propanoate (**5c**). Figure S14: ^1^H NMR methyl 3-(dibutylamino)propanoate (**5d**). Figure S15: ^1^H NMR methyl 3-morpholinopropanoate (**5e**).
