# Supplementary material for: Catalytic Innovations in the Aza-Michael Reaction: An Experimental Benchmarking Focused on Sustainable Approaches
Source: Molecules. 2025 Jun 20;30(13):2674. doi: 10.3390/molecules30132674 (PMC12250736; doi:10.3390/molecules30132674)
Supplement: Supplementary file 1 [file molecules-30-02674-s001.zip › molecules-3595555-supplementary.pdf]

Catalytic Innovations in the Aza-Michael Reaction: An Experimental Benchmarking Focused on Sustainable Approaches

Silvia Izquierdo [1], Carlos J. Durán-Valle [2], Pedro Cintas [2,*] and Ignacio M. López-Coca [1,*]

[1] Department of Organic and Inorganic Chemistry, School of Technology, University Research Institute for Sustainable Territorial Development (INTERRA), Universidad de Extremadura, 10003 Cáceres, Spain; sizquierdo@unex.es
[2] Department of Organic and Inorganic Chemistry, Faculty of Sciences, University Institute for Water Research, Climate Change and Sustainability (IACYS), Universidad de Extremadura, 06006 Badajoz, Spain; carlosdv@unex.es
* Correspondence: pecintas@unex.es (P.C.); iglomar@unex.es (I.M.L.-C.)

SUPPLEMENTARY MATERIALS

For the complete characterization of the hydrothermal carbons HCB and HCC, please refer to Izquierdo et al. 2023 and its ESI; DOI: https://doi.org/10.3390/c9020057 (open-access article under the CC-BY license).

Table of Contents

Solid catalysts characterization 2
- Table S1: K10 porosimetry 2
- Table S2: K10 Energy dispersive X-ray EDX 2
- Table S3: K10 Wavelength-dispersive X-ray fluorescence WDXRF 2
- Table S4: K10 X-ray photoelectron spectroscopy XPS 2
- Table S5: K10 powder X-ray diffractometry PXR 2
- Table S6. Thermogravimetric analysis (TGA) temperature program 3
- Figure S1: K10 SEM images 3
- Figure S2: K10 EDX elemental mapping. 6
- Figure S3: XPS Peak deconvolution for HCB and HCC. 8
- Figure S4. FTIR spectra for chestnut cupule, HCB, and HCC. 8

Product characterization 9
- Figure S5: ^{1}H NMR [Choline][Proline] 9
- Figure S6: ^{1}H NMR 3-(phenylamino)propanenitrile (**4a**) 10
- Figure S7: ^{1}H NMR 3-(benzylamino)propanenitrile (**4b**) 11
- Figure S8: ^{1}H NMR 3-(piperidin-1-yl)propanenitrile (**4c**) 12
- Figure S9: ^{1}H NMR 3-(dibutylamino)propanenitrile (**4d**) 13
- Figure S10: ^{1}H NMR 3-morpholinopropanenitrile (**4e**) 14
- Figure S11: ^{1}H NMR methyl 3-(phenylamino)propanoate (**5a**) 15
- Figure S12: ^{1}H NMR methyl 3-(benzylamino)propanoate (**5b**) 16
- Figure S13: ^{1}H NMR methyl 3-(piperidin-1-yl)propanoate (**5c**) 17
- Figure S14: ^{1}H NMR methyl 3-(dibutylamino)propanoate (**5d**) 18
- Figure S15: ^{1}H NMR methyl 3-morpholinopropanoate (**5e**) 19

Solid catalysts characterization

Table S1: K10 porosimetry

Surface area[a]	V_{micro}[a]	V_{meso}[b]	V_{macro}[b]	V_{total}
$m^2\ g^{-1}$		$cm^3\ g^{-1}$		
279.5	0.107	0.0787	0.7804	0.9661

[a] BET method. [b] Mercury porosimetry.

Table S2: K10 Energy dispersive X-ray EDX

**C**	**O**	**Na**	**Mg**	**Al**	**Si**	**K**	**Ca**	**Ti**	**Fe**
8.75	48.61	0.17	1.07	7.38	32.2	1.39	0.62	0.35	2.56

Table S3: K10 Wavelength-dispersive X-ray fluorescence WDXRF

Oxide	Concentration, %
SiO_2	67.36
Al_2O_3	12.10
Fe_2O_3	3.05
K_2O	1.70
MgO	1.37
TiO_2	0.537
Na_2O	0.29
CaO	0.221
ZrO_2	0.0338
CuO	0.0131
ZnO	0.0106

Table S4: K10 X-ray photoelectron spectroscopy XPS

C	N	O	Mg	Al	Si	Fe
7.13	0.41	51.58	2.10	5.25	30.92	2.61

Table S5: K10 powder X-ray diffractometry PXR

2 theta	intensity	%
5.77	4405	72.02
8.89	4054	66.29
17.81	1538	25.15
19.85	2063	33.73
20.83	2004	32.77
26.63	6116	100.00
26.83	2123	34.71
27.95	1126	18.41
35.04	1092	17.85
36.55	1009	16.50
39.45	758	12.39
42.47	813	13.29
45.49	851	13.91

50.13	854	13.96
59.95	754	12.33

Table S6. Thermogravimetric analysis (TGA) temperature program

Process	Initial temperature, ºC	Final temperature, ºC	Carrier gas	Time, min
Stabilization	40	40	Ar	30
Heating	40	105	Ar	7.5
Isothermic	105	105	Ar	10
Heating	105	900	Ar	26.5
Isothermic	900	900	Ar	7
Isothermic	900	900	Air	30

Figure S1: K10 SEM images

a) 500×

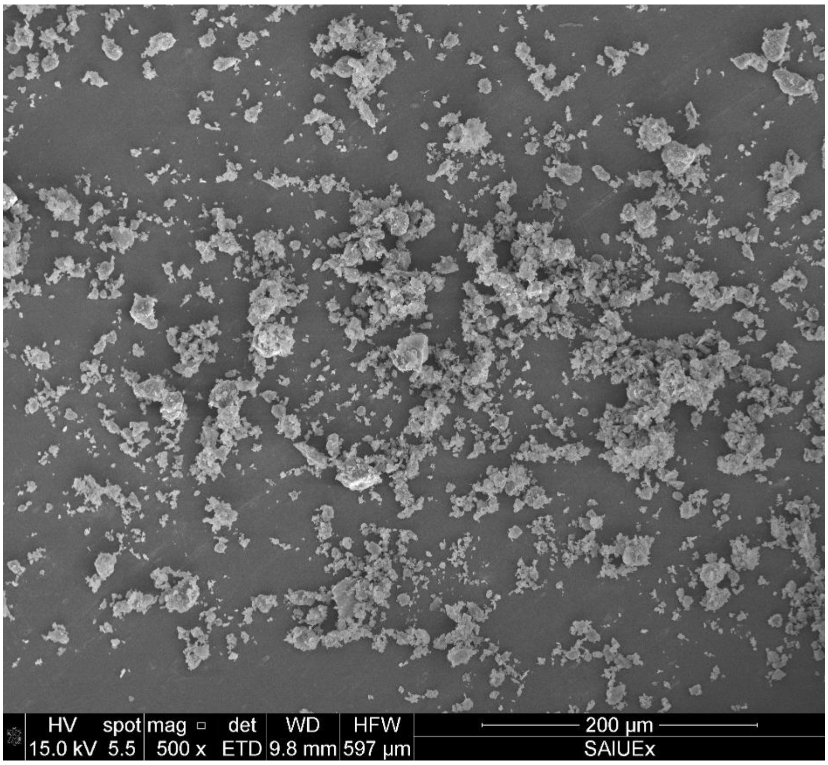

b) 2500×

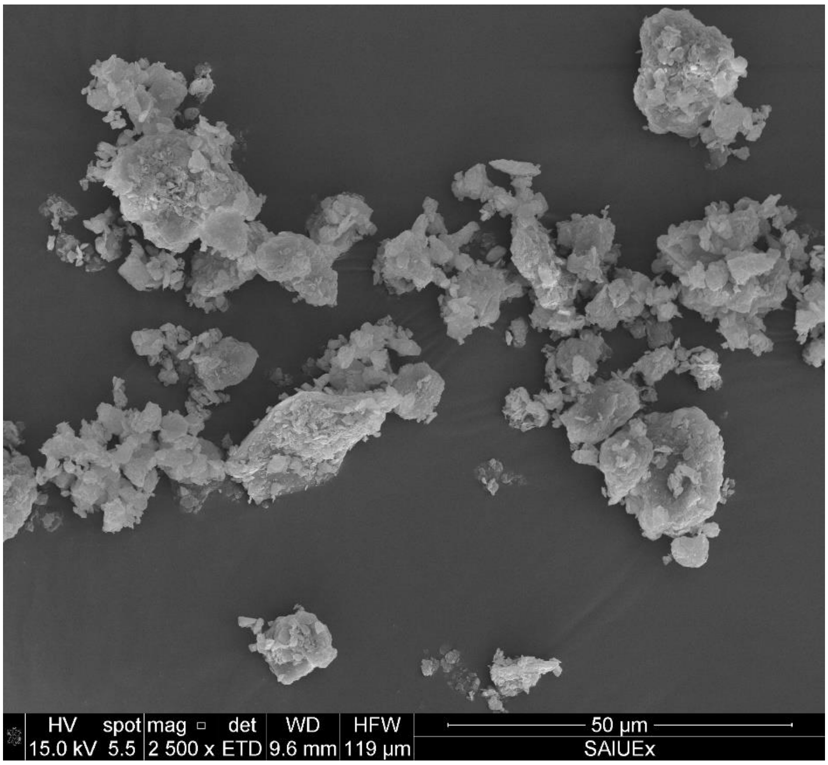
HV
15.0 kV
spot
5.5
mag
2 500 x
det
ETD
WD
9.6 mm
HFW
119 µm
50 µm
SAIUEx

c) 3500×

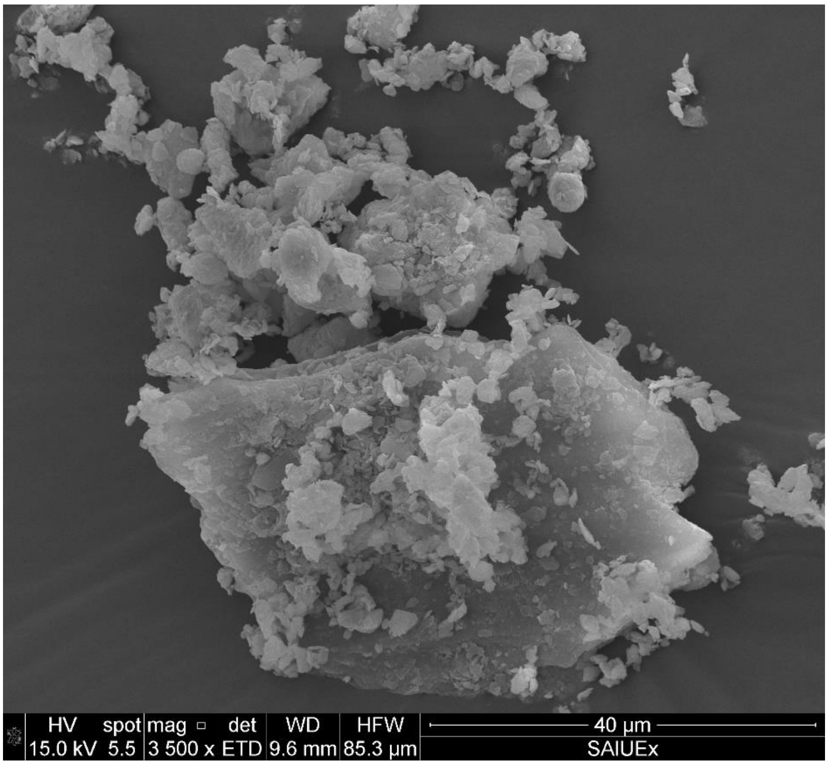
HV
15.0 kV
spot
5.5
mag
3 500 x
det
ETD
WD
9.6 mm
HFW
85.3 µm
40 µm
SAIUEx

d) 5000×

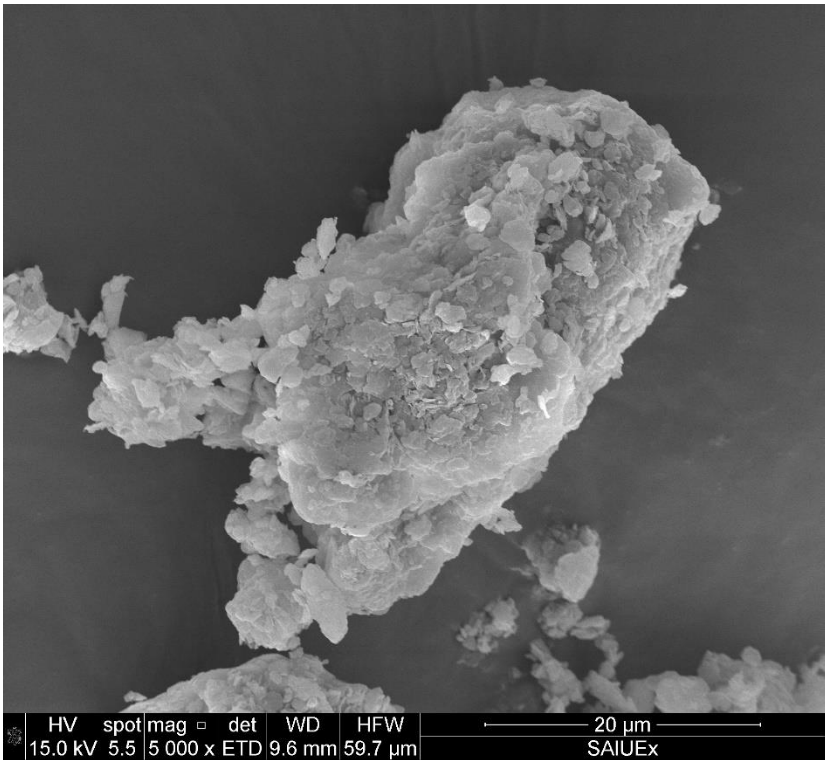
HV 15.0 kV
spot 5.5
mag 5 000 x
det ETD
WD 9.6 mm
HFW 59.7 µm
20 µm
SAIUEx

e) 8000×

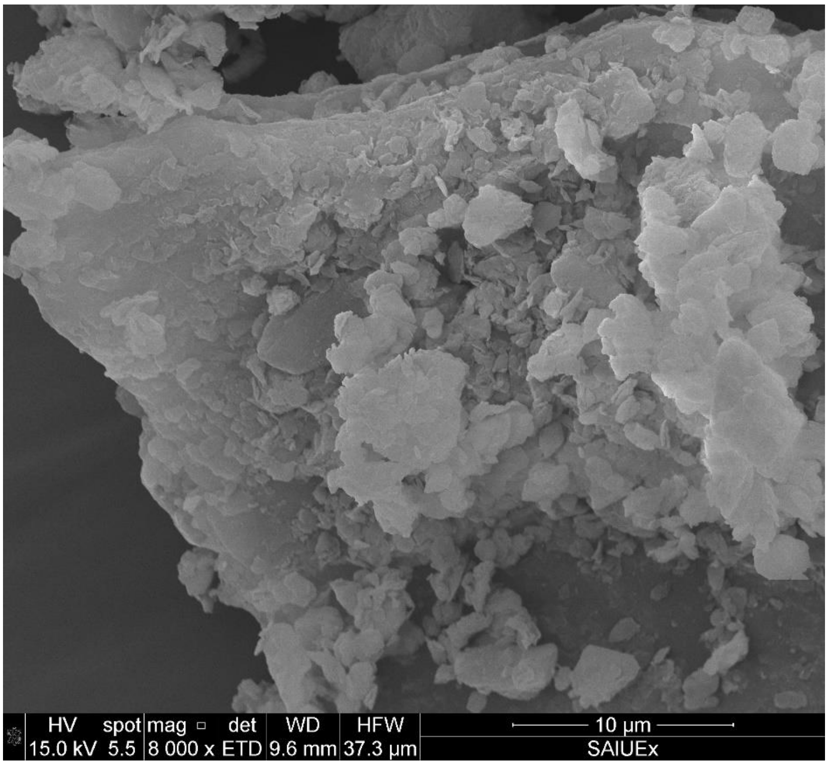
HV 15.0 kV
spot 5.5
mag 8 000 x
det ETD
WD 9.6 mm
HFW 37.3 µm
10 µm
SAIUEx

Figure S2: K10 EDX elemental mapping.

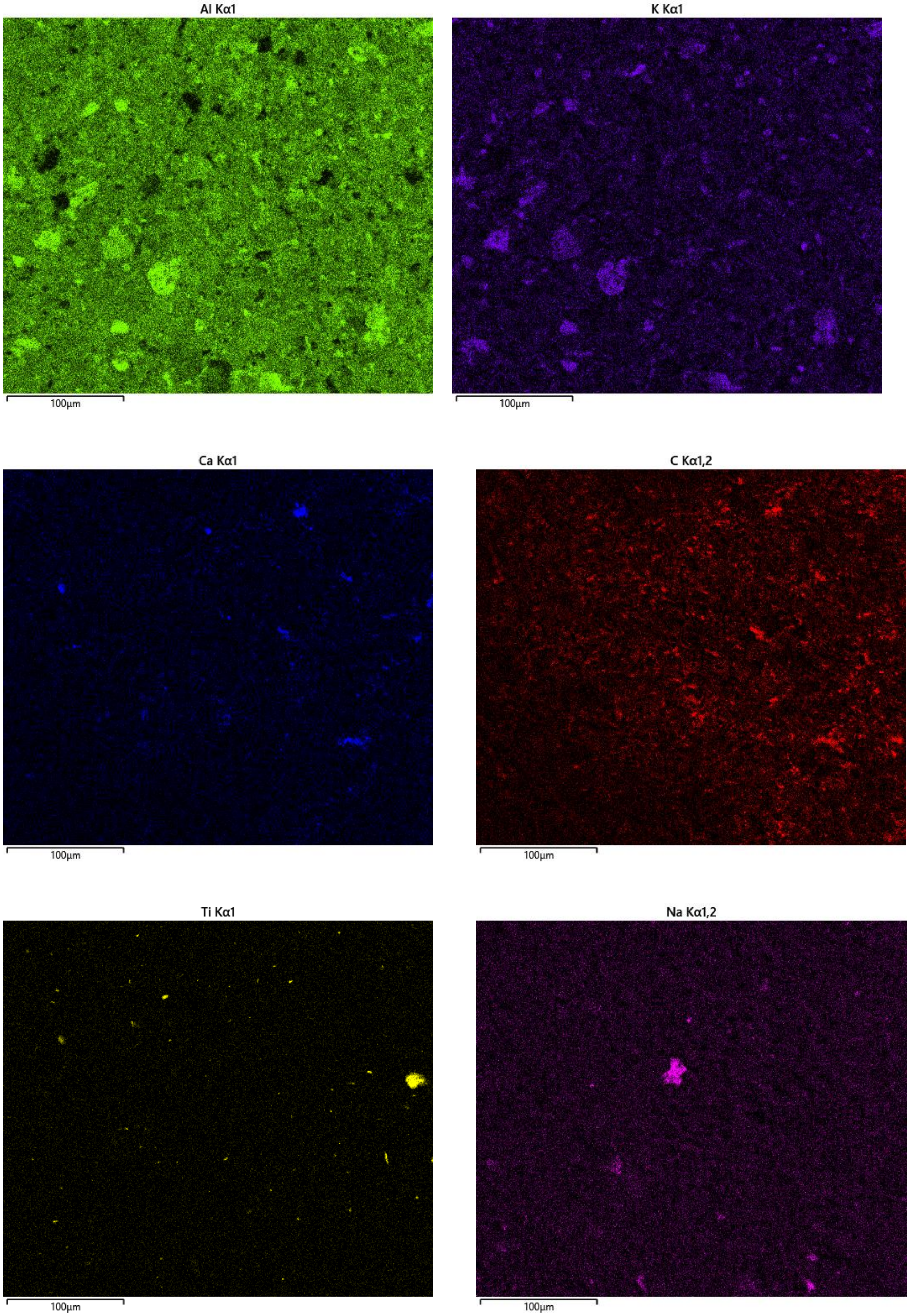

Fe Kα1

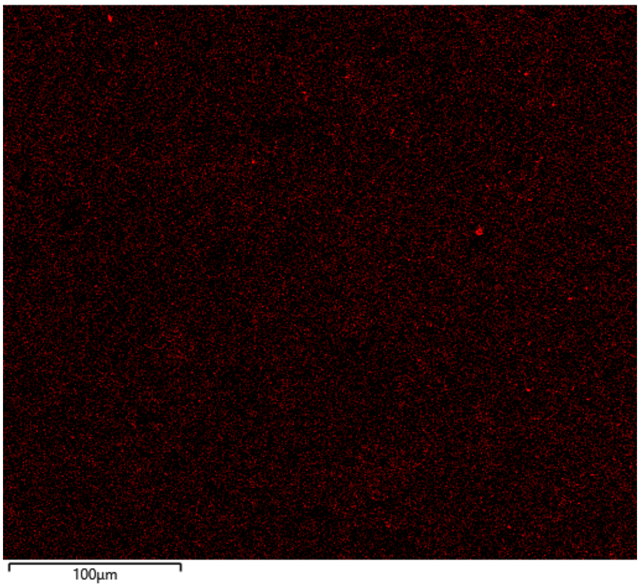

O Kα1

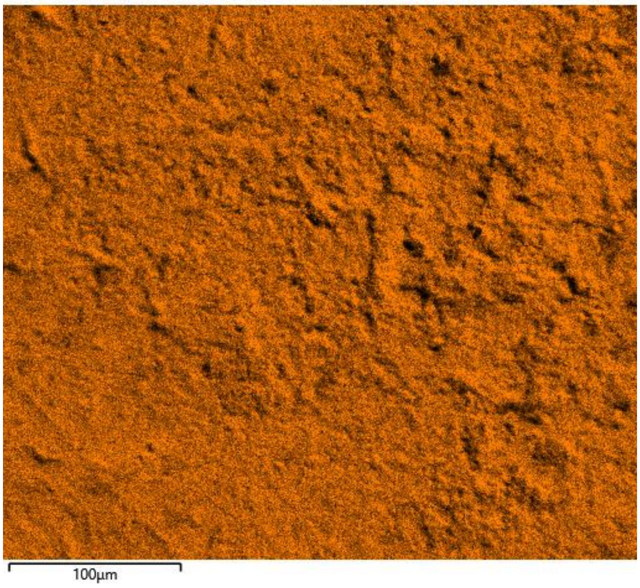

Mg Kα1,2

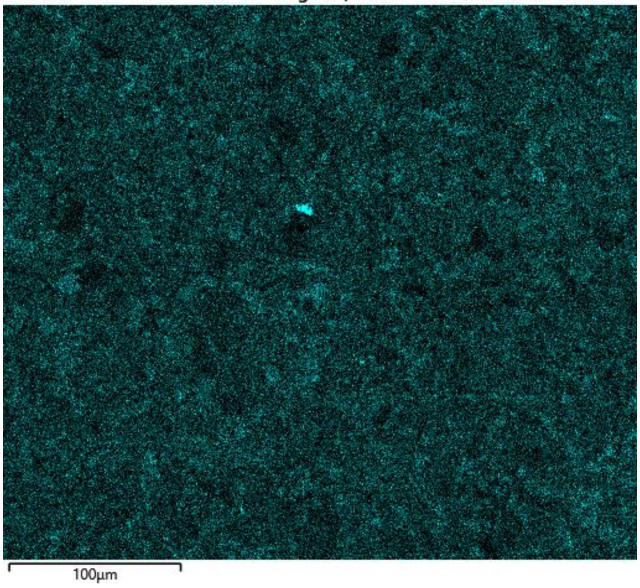

Si Kα1

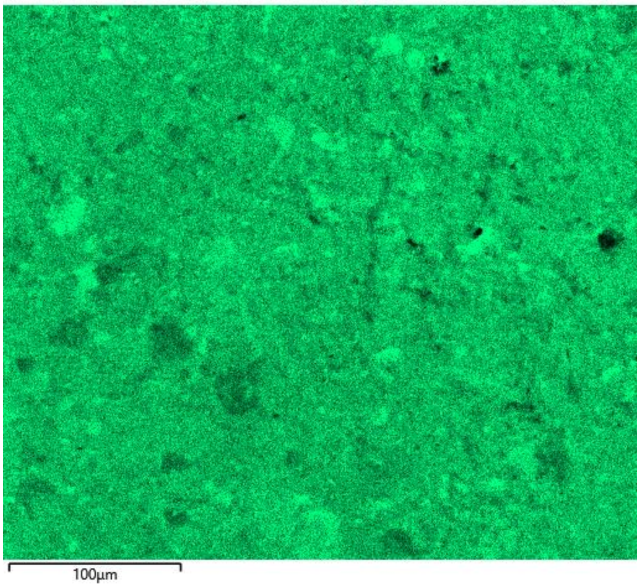

Figure S3: XPS Peak deconvolution for HCB and HCC.

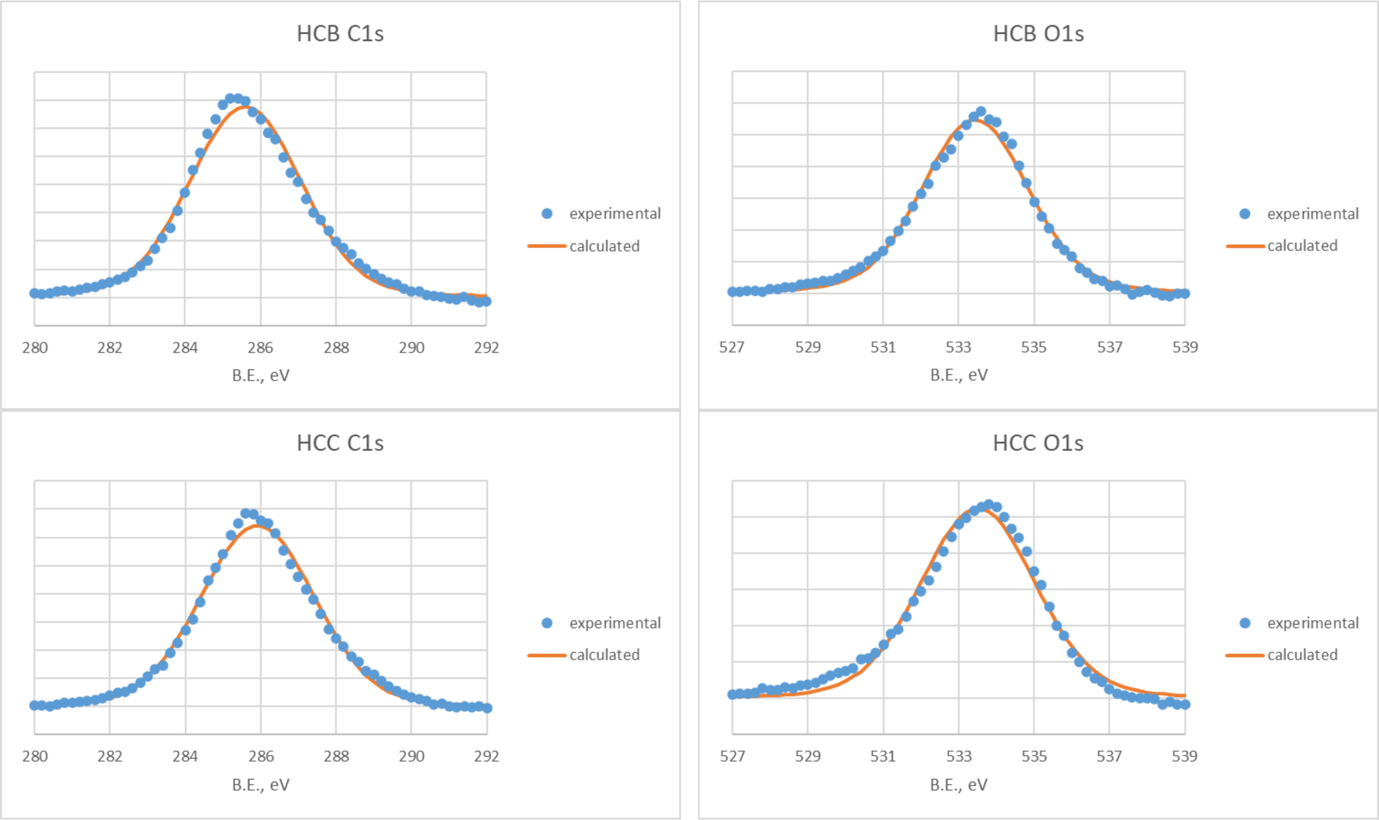

Figure S4. FTIR spectra for chestnut cupule, HCB, and HCC.

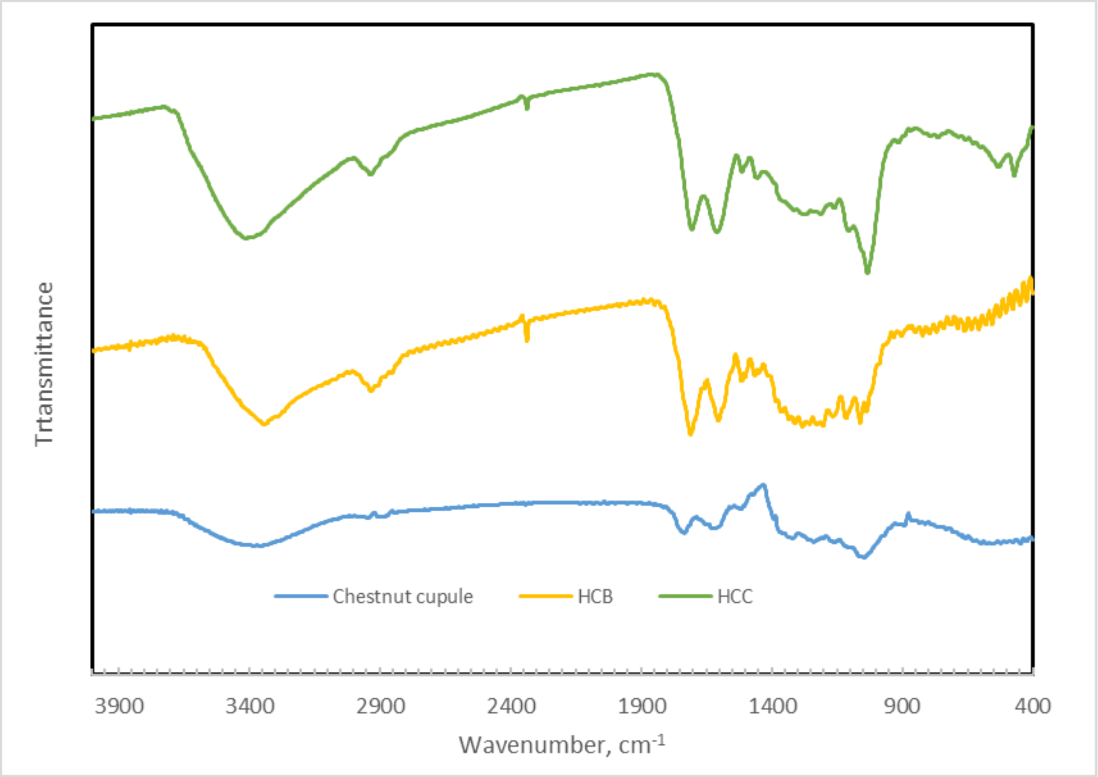

Product characterization

Figure S5: ^1H NMR [Choline][Proline]

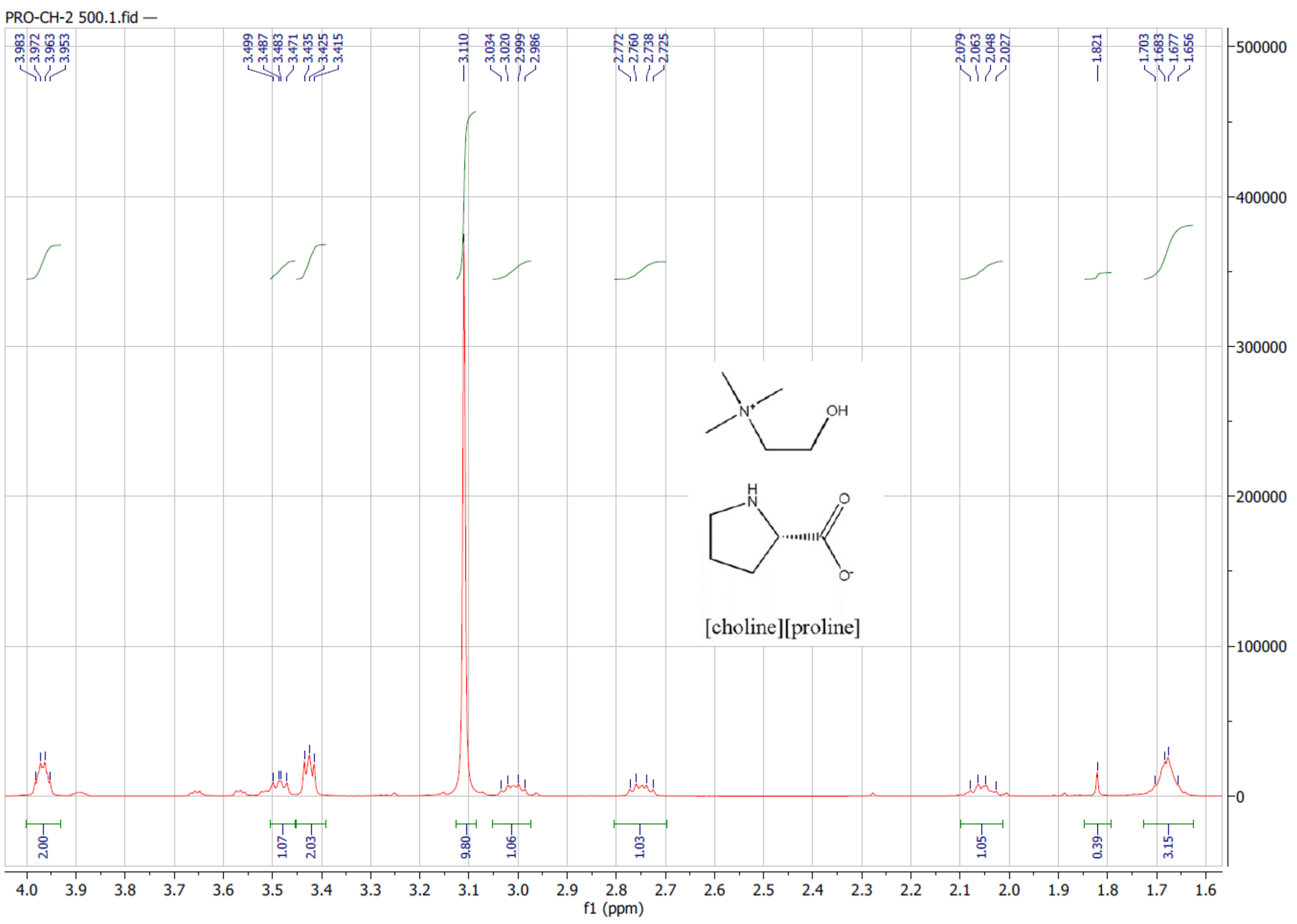

[Choline][Proline]

^1H NMR (500.15 MHz, $CDCl_3$): δ_H 3.98–3.95 (m, 2H, CH_2-OH), 3.50–3.47 (m, 1H, CH-COO^-), 3.44 (t, J_{HH} 5 Hz, 2H, CH_2-NMe_3), 3.11 (s, 9H, NMe_3), 3.03–2.99 (m, 1H, CHH′-NH), 2.77–2.73 (m, 1H, CHH′-NH-NH), 2.08–2.03 (m, 1H, CHH′-$CHCOO^-$), 1.82 (as, 1H, NH), 1.70–1.66 (m, 3H, $CH_2CH_2CH_2$, CHH′-$CHCOO^-$)

Figure S6: ^{1}H NMR 3-(phenylamino)propanenitrile (**4a**)

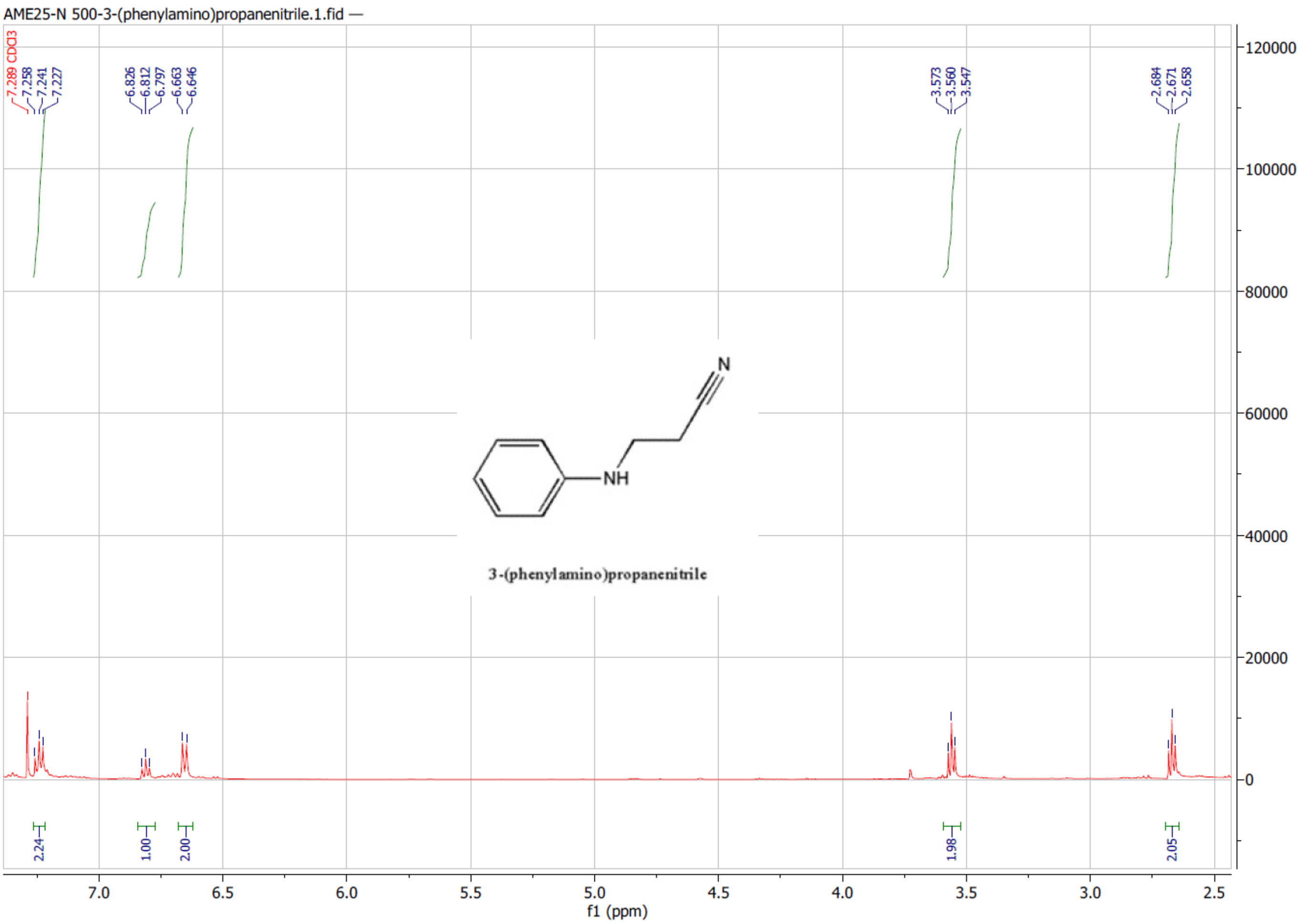

3-(Phenylamino)propanenitrile (**4a**)

Yellow liquid. ^{1}H NMR (500.15 MHz, $CDCl_3$): δ_H 7.26–7.23 (m, 2H, arom), 6.83 (t, J_{HH} 7 Hz, 1H, arom), 6.66 (d, J_{HH} 8.5, 2H, arom), 3.57 (t, J_{HH} 6.5 Hz, 2H, $NHCH_2$), 2.68 (t, J_{HH} 6.5 Hz, 2H, C<u>H</u>$_2$CN).

Figure S7: ^{1}H NMR 3-(benzylamino)propanenitrile (**4b**)

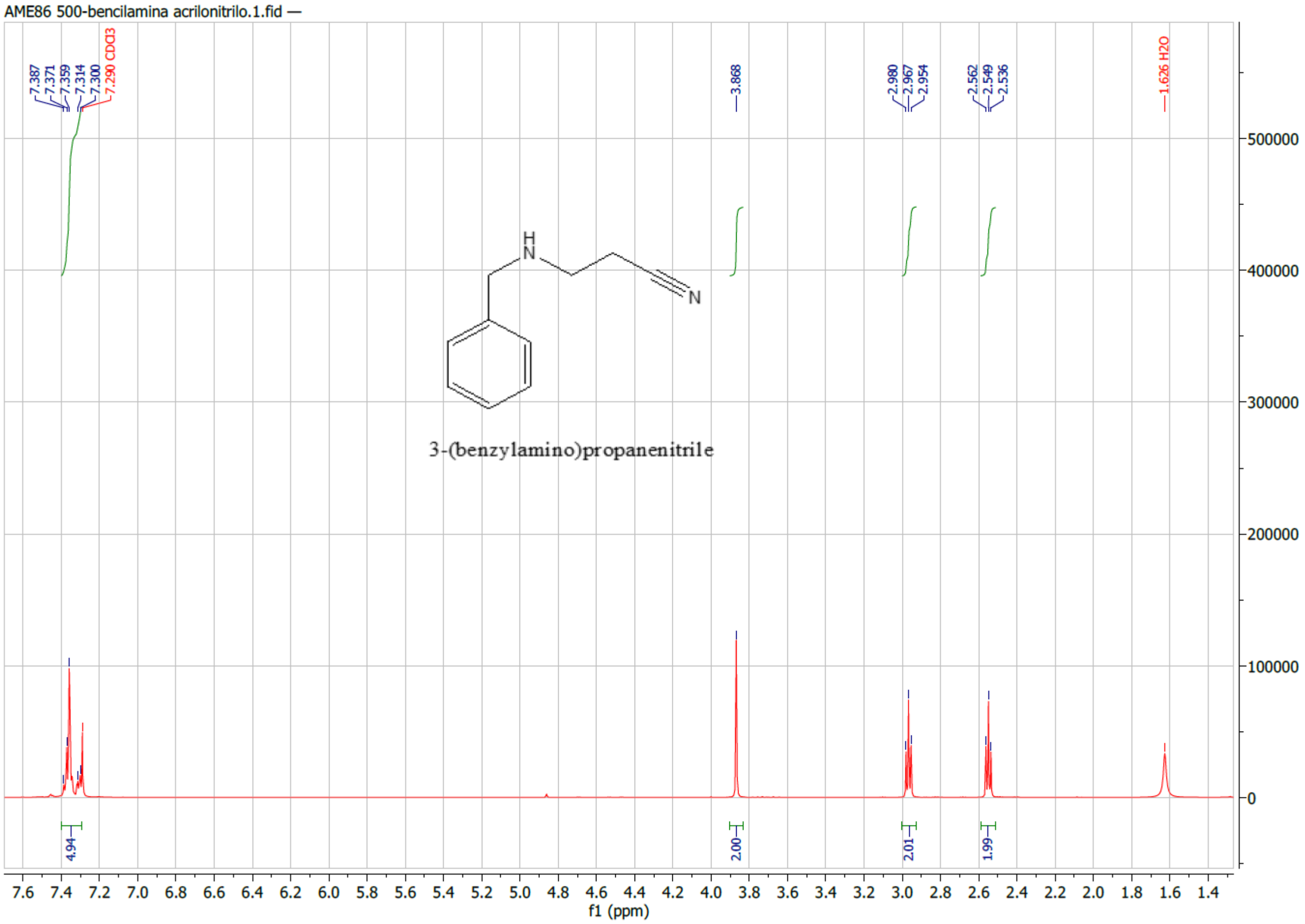

3-(Benzylamino)propanenitrile (**4b**)

Pale yellow liquid. ^{1}H NMR (500.15 MHz, $CDCl_3$): δ_H 7.39–7.30 (m, 5H, arom), 3.87 (s, 2H, Ph-CH_2-NH), 2.98 (d, J_{HH} 6.5 Hz, 2H, NH-C<u>H</u>$_2$-CH_2), 2.56 (d, J_{HH} 6.5 Hz, 2H, NH-CH_2-C<u>H</u>$_2$).

Figure S8: ^{1}H NMR 3-(piperidin-1-yl)propanenitrile (**4c**)

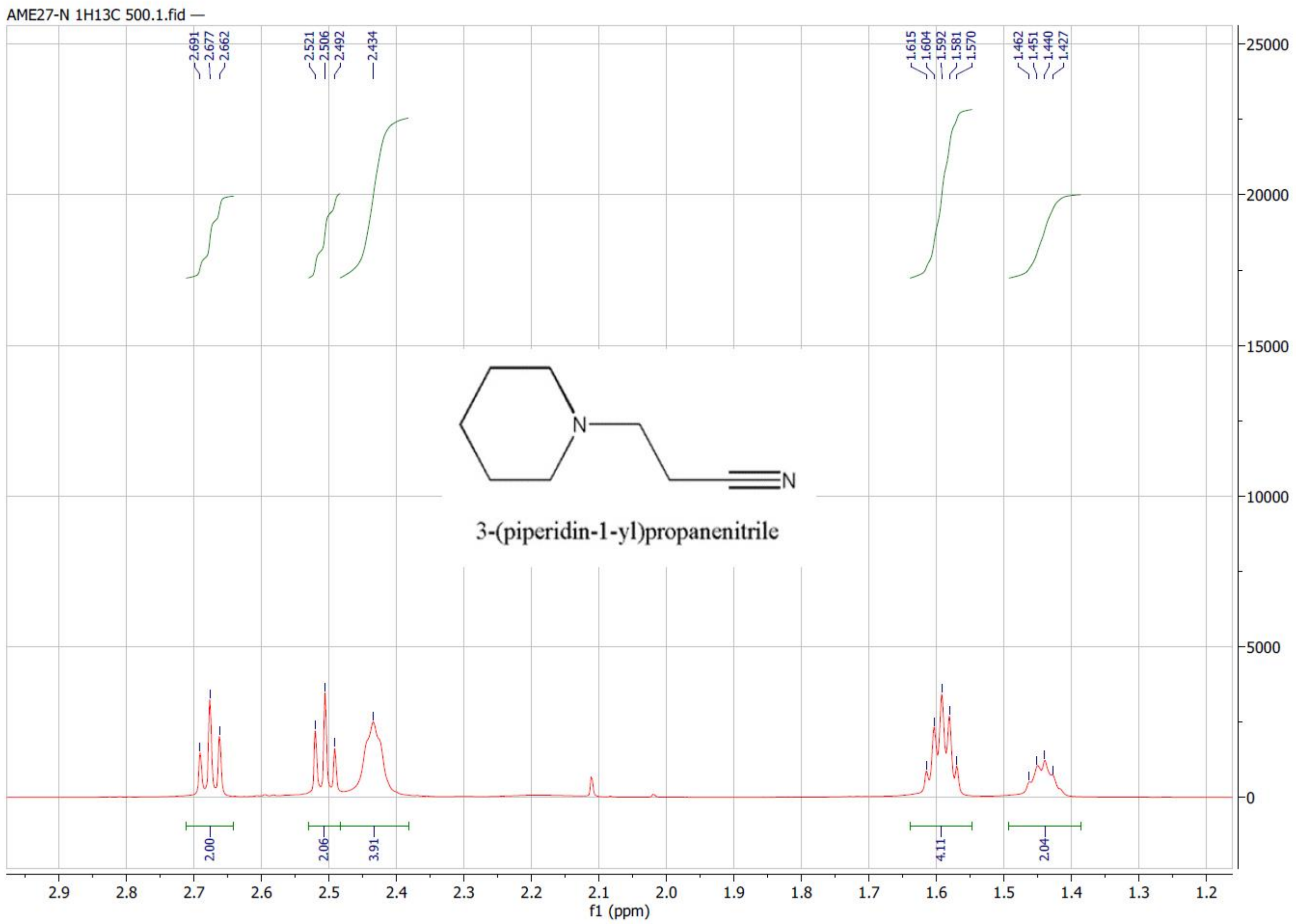

3-(Piperidin-1-yl)propanenitrile (**4c**)

Yellow liquid. ^{1}H NMR (500.15 MHz, $CDCl_3$): δ_H 2.69 (t, J_{HH} 7 Hz, 2H, NC$\underline{H_2}$$CH_2$CN), 2.52 (t, J_{HH} 7.5 Hz, 2H, CH_2CN), 2.43 (bs, 4H, CH_2NCH_2), 1.62–1.57 (m, 4H, 2CH_2), 1.46–1.43 (m, 2H, CH_2).

Figure S9: ^{1}H NMR 3-(dibutylamino)propanenitrile (**4d**)

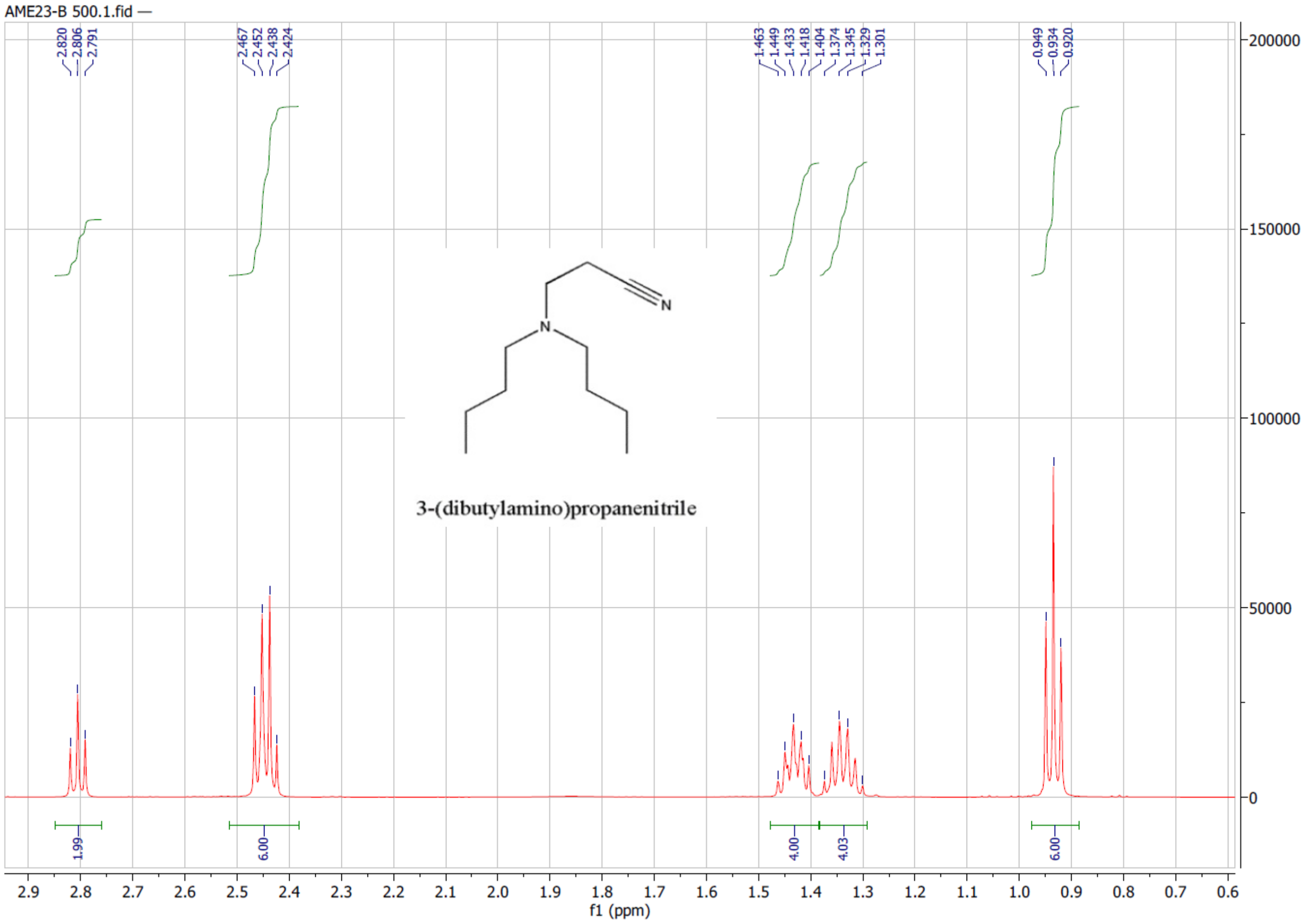

3-(Dibutylamino)propanenitrile (**4d**)

Pale yellow liquid. ^{1}H NMR (500.15 MHz, $CDCl_3$): δ_H 2.82 (t, J_{HH} 7 Hz, 2H, N-C<u>H</u>$_2$$CH_2$CN), 2.47–2.42 (m, 6H, (C<u>H</u>$_2$)$_2$-N-CH_2C<u>H</u>$_2$CN), 1.46 (qu, J_{HH} 8 Hz, 4H, N(CH_2C<u>H</u>$_2$$CH_2$$CH_3$)$_2$, 1.37 (qu, J_{HH} 8 Hz, 4H, N($CH_2$$CH_2$C<u>H</u>$_2$$CH_3$)$_2$, 0.95 (t, J_{HH} 7.5 Hz, 6H, 2CH_3).

Figure S10: ^{1}H NMR 3-morpholinopropanenitrile (**4e**)

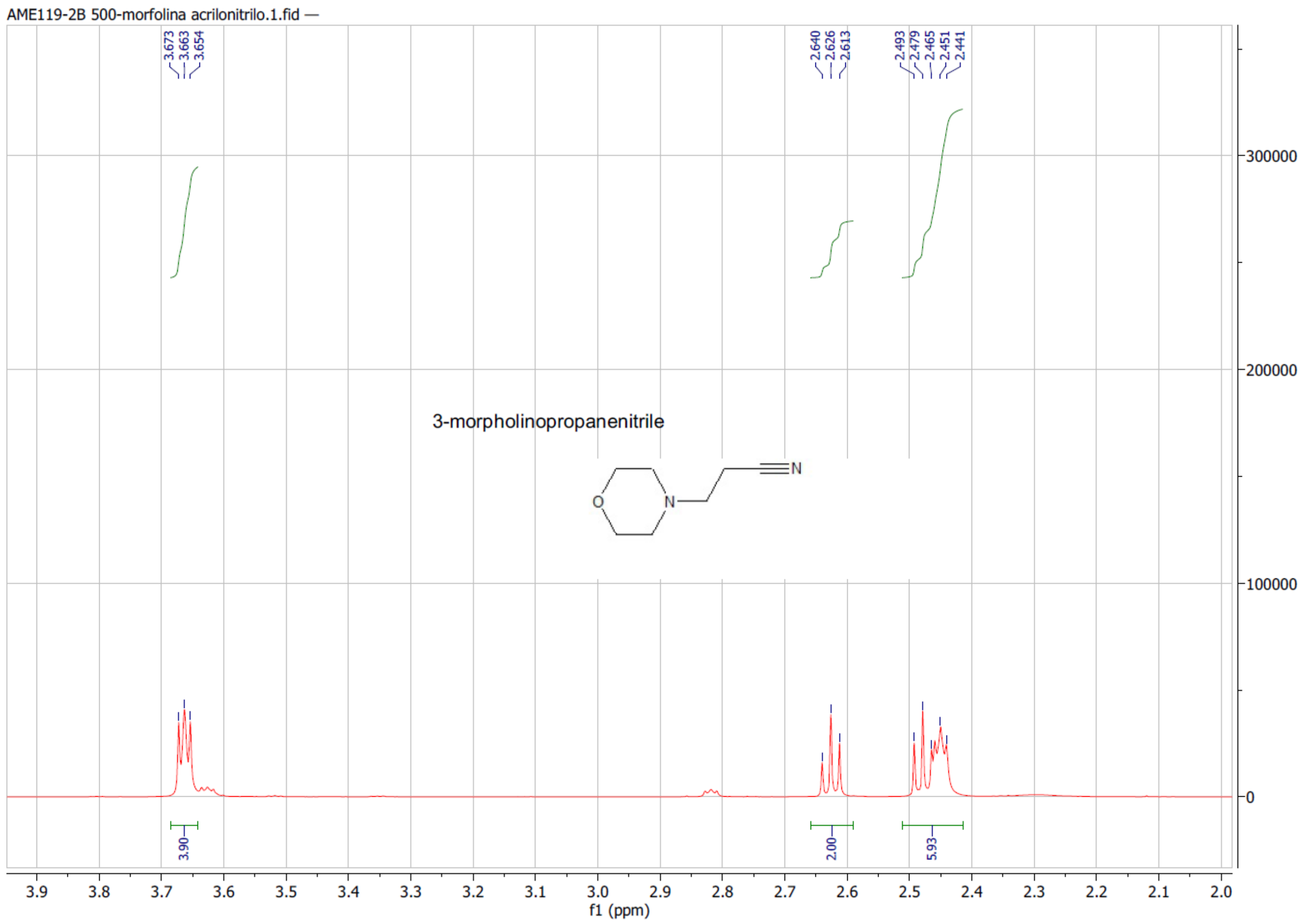

3-Morpholinopropanenitrile (**4e**)

Pale yellow liquid. ^{1}H NMR (500.15 MHz, $CDCl_3$): δ_H 3.67 (t, J_{HH} 5 Hz, 4H, CH_2OCH_2), 2.64 (t, J_{HH} 7 Hz, 2H, N-C<u>H</u>$_2$$CH_2$CN), 2.49–2.44 (m, 6H, (C<u>H</u>$_2$)$_2$-N-CH_2C<u>H</u>$_2$CN).

Figure S11: ^{1}H NMR methyl 3-(phenylamino)propanoate (**5a**)

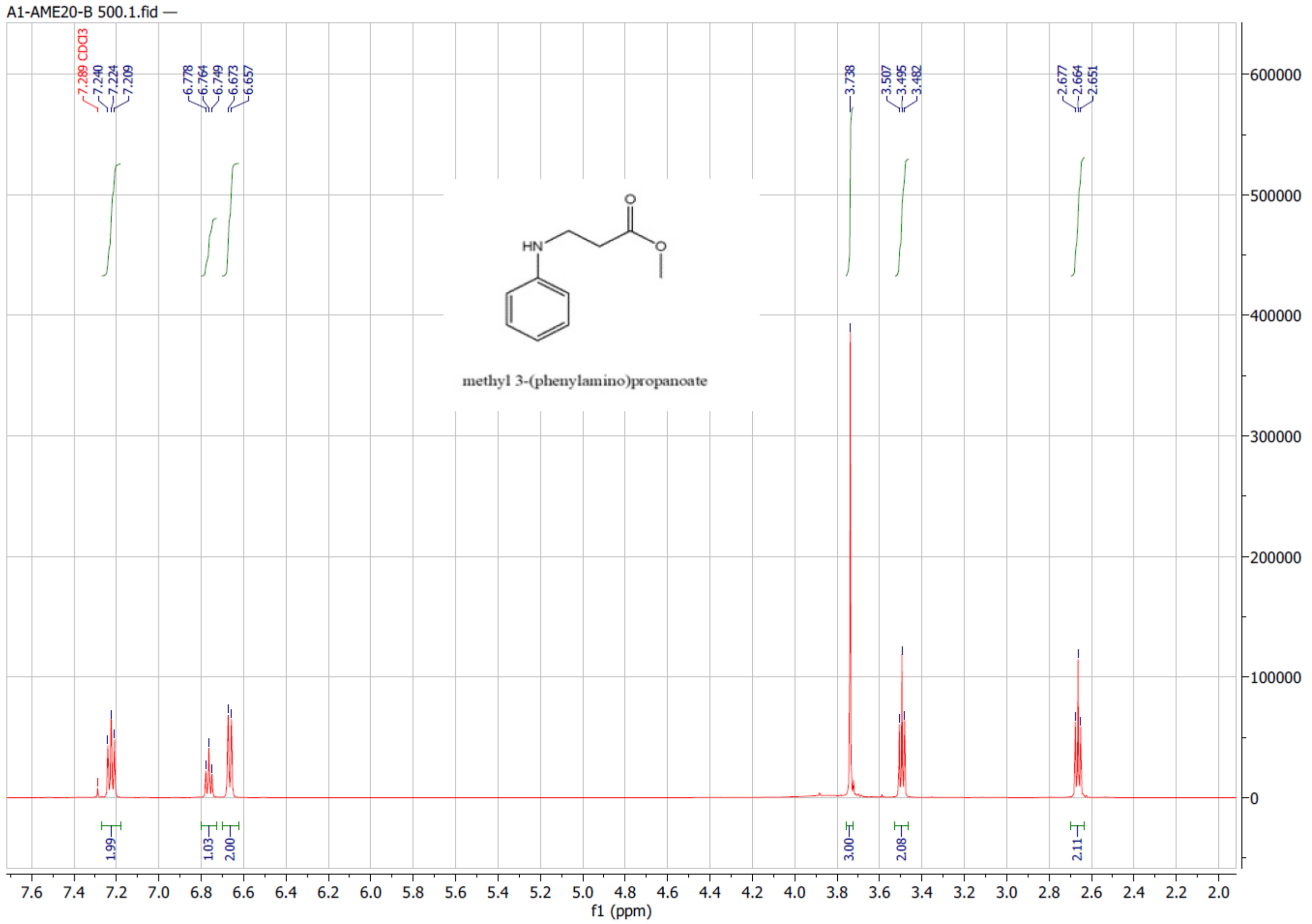

Methyl 3-(phenylamino)propanoate (**5a**)

Yellow liquid. ^{1}H NMR (500.15 MHz, $CDCl_3$): δ_H 7.24 (t, J_{HH} 8 Hz, 2H, *m*-arom), 6.78 (t, J_{HH} 7 Hz, 1H, *p*-arom), 6.67 (d, J_{HH} 8 Hz, 2H, *o*-arom), 3.74 (s, 3H, OCH_3), 3.51 (t, J_{HH} 6 Hz, 2H, $NHCH_2$), 2.68 (t, J_{HH} 6.5 Hz, 2H, $C\underline{H_2}CO_2CH_3$).

Figure S12: ^{1}H NMR methyl 3-(benzylamino)propanoate (**5b**)

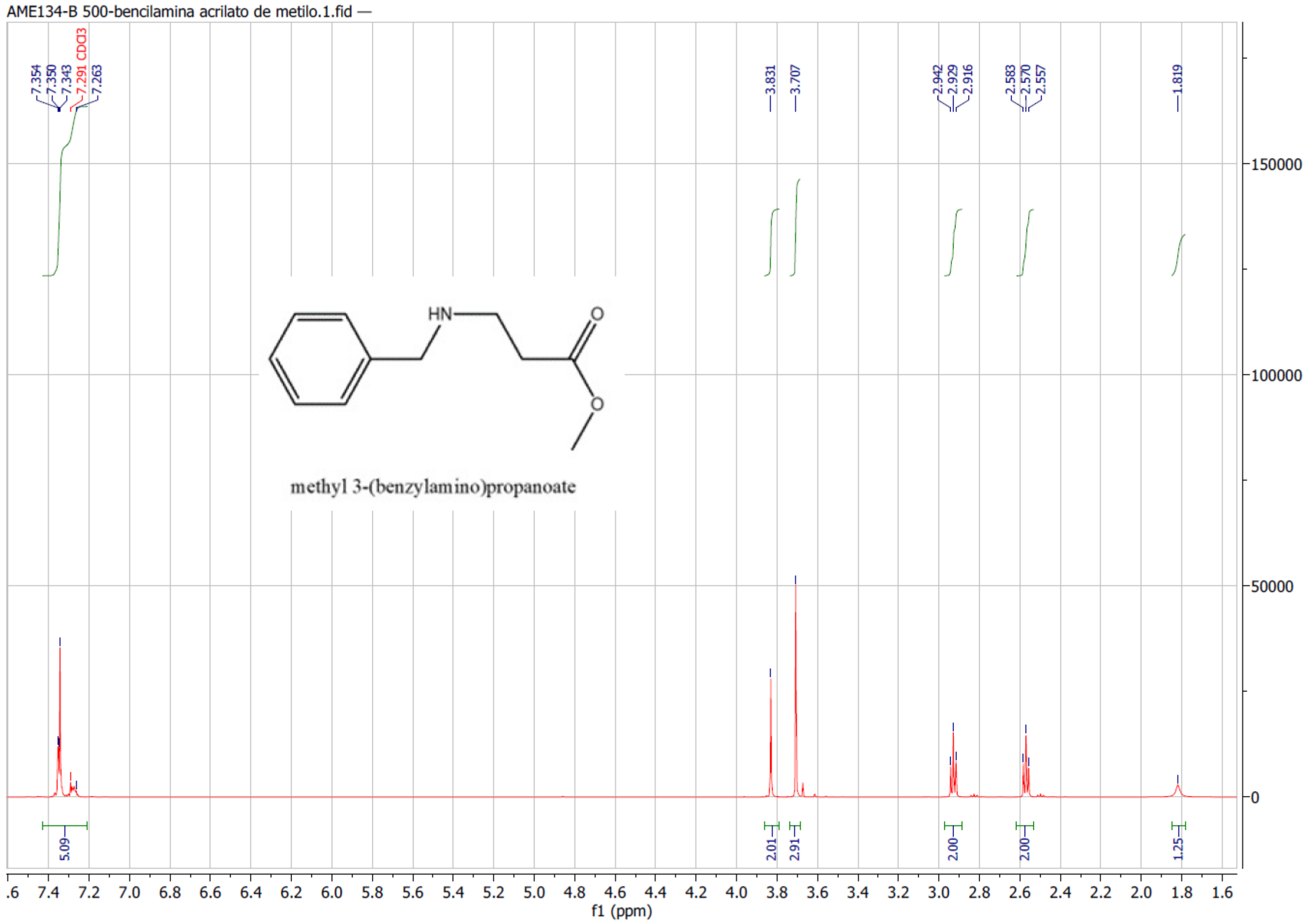

Methyl 3-(benzylamino)propanoate (**5b**)

Pale yellow liquid. ^{1}H NMR (500.15 MHz, $CDCl_3$): δ_H 7.35–7.26 (m, 5H, arom), 3.83 (s, 2H, Ph-CH$_2$-NH), 3.71 (s, 3H, OCH_3), 2.94 (d, J_{HH} 6.5 Hz, 2H, NH-C$\underline{H_2}$-CH$_2$), 2.58 (d, J_{HH} 6.5 Hz, 2H, NH-CH$_2$-C$\underline{H_2}$), 1.82 (bs, 1H, NH).

Figure S13: ^{1}H NMR methyl 3-(piperidin-1-yl)propanoate (**5c**)

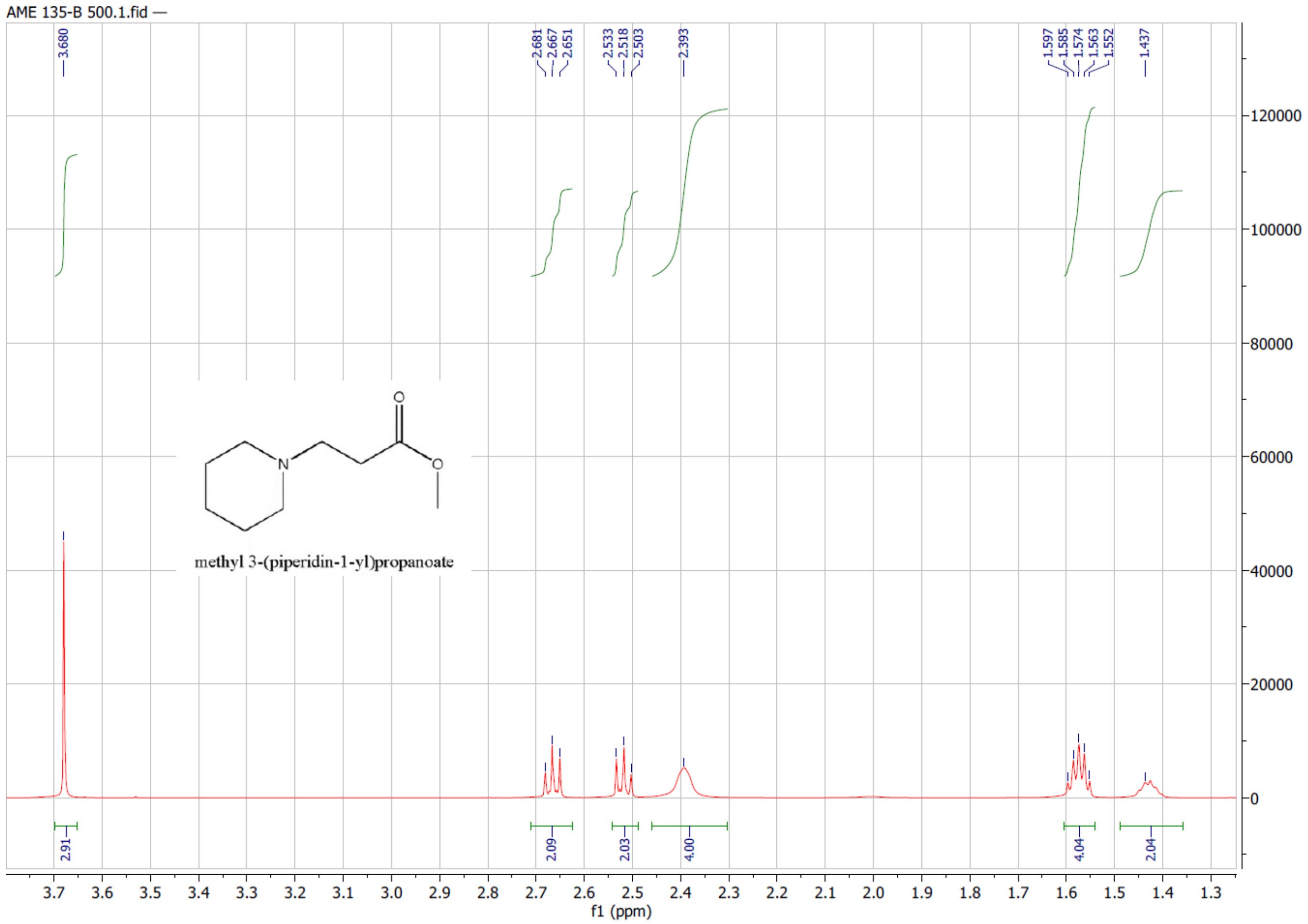

Methyl 3-(piperidin-1-yl)propanoate (**5c**)

Yellow liquid. ^{1}H NMR (500.15 MHz, $CDCl_3$): δ_H 3.68 (s, 3H, CH_3), 2.68 (t, J_{HH} 7 Hz, 2H, NC$\underline{H_2}$$CH_2CO_2CH_3$), 2.53 (t, J_{HH} 7.5 Hz, 2H, C$\underline{H_2}$$CO_2CH_3$), 2.39 (bs, 4H, CH_2NCH_2), 1.60 (qu, J_{HH} 5.5 Hz, 4H, 2CH_2), 1.42 (m, 2H, CH_2).

Figure S14: ^{1}H NMR methyl 3-(dibutylamino)propanoate (**5d**)

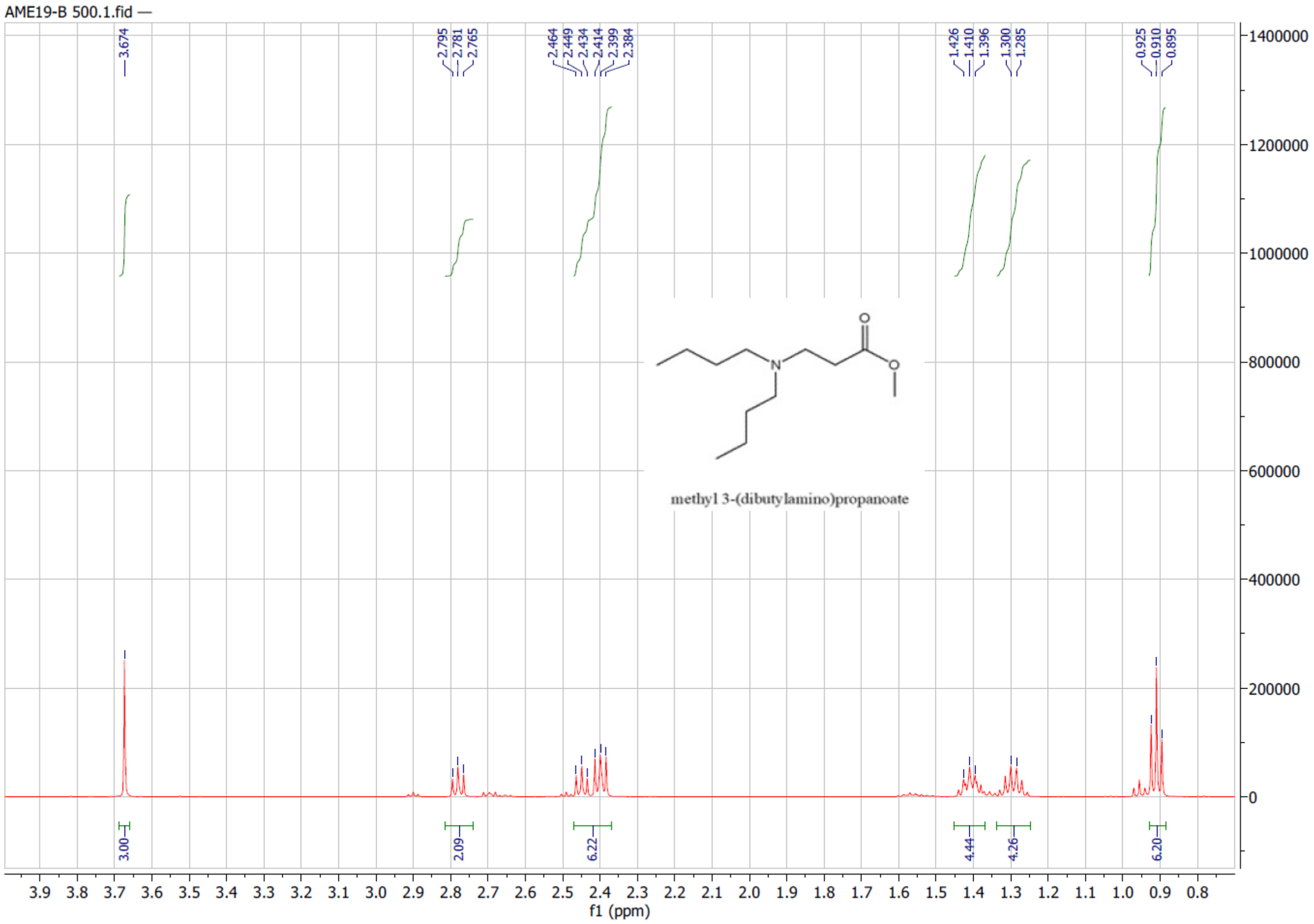

Methyl 3-(dibutylamino)propanoate (**5d**)

Pale yellow liquid.^{1}H NMR (500.15 MHz, $CDCl_3$): δ_H 3.67 (s, 3H, OCH_3), 2.80 (t, J_{HH} 7 Hz, 2H, N-C<u>H2</u>$CH_2CO_2CH_3$), 2.46 (t, J_{HH} 7.5 Hz, 2H, C<u>H2</u>CO_2CH_3), 2.41 (t, J_{HH} 7.5 Hz, 4H, (C<u>H2</u>)$_2$-N-$CH_2CH_2COOCH_3$), 1.43 (qu, J_{HH} 8 Hz, 4H, N(CH_2C<u>H2</u>CH_2CH_3)$_2$), 1.30 (sext, J_{HH} 7.5 Hz, 4H, N(CH_2CH_2C<u>H2</u>CH_3)$_2$), 0.93 (t, J_{HH} 7.5 Hz, 6H, $2CH_3$).

Figure S15: ^{1}H NMR methyl 3-morpholinopropanoate (**5e**)

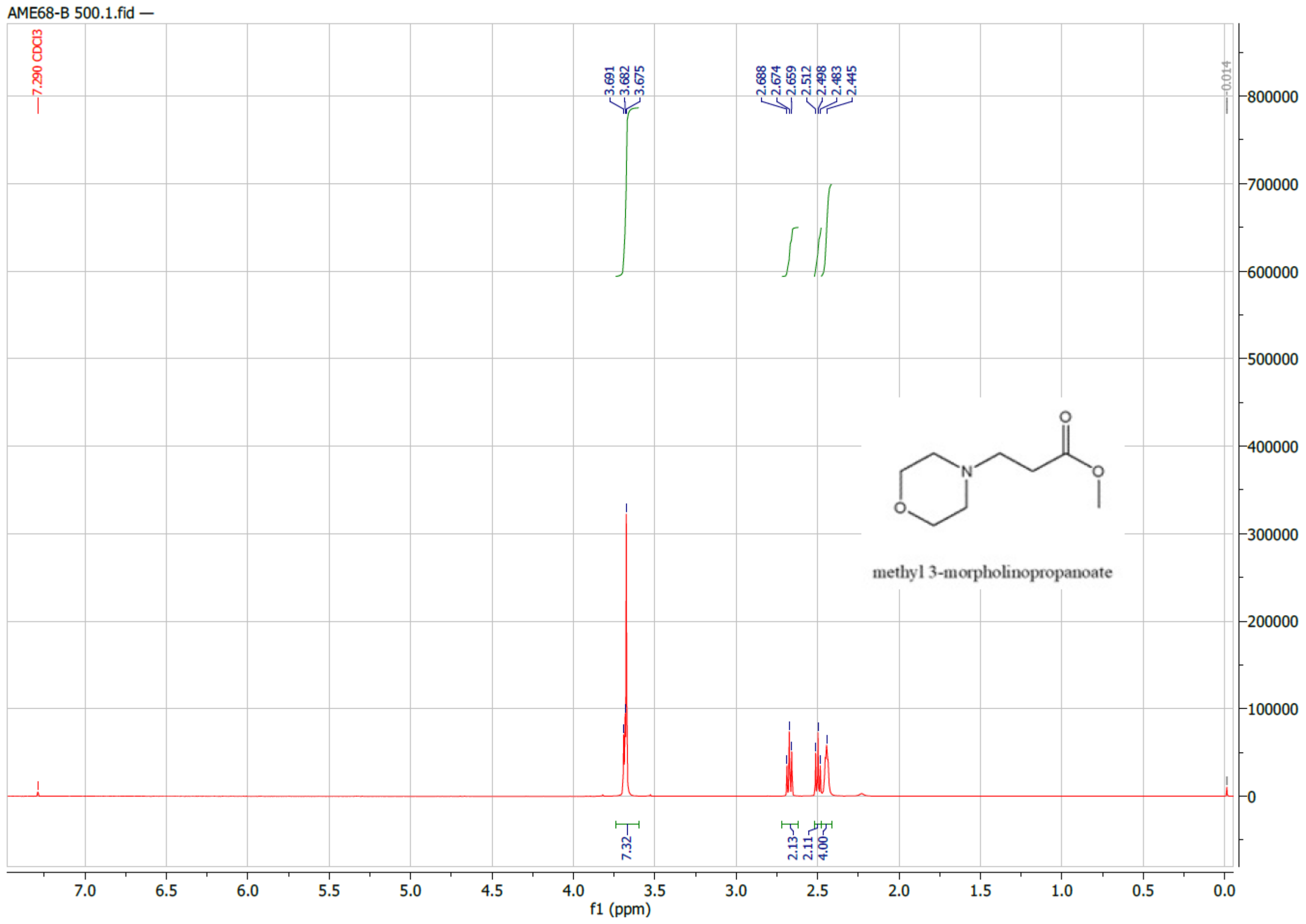

Methyl 3-morpholinopropanoate (**5e**)

Pale yellow liquid. ^{1}H NMR (500.15 MHz, $CDCl_3$): δ_H 3.69–3.68 (m, 7H, CH_2OCH_2, OCH_3), 2.69 (t, J_{HH} 7 Hz, 2H, N-$C\underline{H_2}CH_2CO_2CH_3$), 2.51 (t, J_{HH} 7 Hz, 2H, N-$CH_2C\underline{H_2}CO_2CH_3$), 2.45 (bs, 4H, $(C\underline{H_2})_2$-N-$CH_2CH_2COOCH_3$).
